# First-Trimester Gestational Diabetes Mellitus Risk Prediction with Machine Learning Techniques: Results from the BORN2020 Cohort Study

**DOI:** 10.3390/jcm15062461

**Published:** 2026-03-23

**Authors:** Nikolaos Pazaras, Antonios Siargkas, Antigoni Tranidou, Aikaterini Apostolopoulou, Ioannis Tsakiridis, Panagiotis D. Bamidis, Sofoklis Stavros, Anastasios Potiris, Michail Chourdakis, Themistoklis Dagklis

**Affiliations:** 1 Third Department of Obstetrics and Gynecology, School of Medicine, Faculty of Health Sciences, Aristotle University of Thessaloniki, 541 24 Thessaloniki, Greece; 2 Laboratory of Hygiene, Social & Preventive Medicine and Medical Statistics, School of Medicine, Faculty of Health Sciences, Aristotle University of Thessaloniki, 541 24 Thessaloniki, Greece; 3 Laboratory of Medical Physics and Digital Innovation, School of Medicine, Faculty of Health Sciences, Aristotle University of Thessaloniki (AUTH), 541 24 Thessaloniki, Greece; 4 Third Department of Obstetrics and Gynecology, University General Hospital “ATTIKON”, Medical School, National and Kapodistrian University of Athens, 124 62 Athens, Greece

**Keywords:** machine learning, ML, gradient boosting methods, SHAP, explainable AI, class imbalance, gestational diabetes mellitus, prediction, GDM, maternal nutrition, micronutrients

## Abstract

**Background**: Gestational diabetes mellitus (GDM) affects many pregnancies worldwide and is associated with adverse maternal and fetal outcomes. Current screening at 24–28 weeks limits opportunities for early intervention. We evaluated whether machine learning (ML) models using first-trimester clinical and dietary data can predict GDM risk before the standard oral glucose tolerance test. **Methods**: We analyzed data from 797 pregnant women enrolled in the BORN2020 prospective cohort study (Thessaloniki, Greece). Ten ML algorithms were evaluated across five class-imbalance handling strategies using stratified 5-fold cross-validation, with final evaluation on an independent 20% held-out test set. Features included maternal demographics, obstetric history, lifestyle factors, and 22 dietary micronutrient intakes from the pre-pregnancy period assessed by Food Frequency Questionnaire. **Results**: The best-performing model (Logistic Regression without resampling) achieved an AUC-ROC of 0.664 (95% CI: 0.542–0.777), with sensitivity of 0.783 and NPV of 0.932 at the pre-specified threshold. The high NPV should be interpreted in the context of the low GDM prevalence (14.7%), as NPV is mathematically dependent on disease prevalence. A reduced nine-feature model using only routine clinical and demographic variables achieved a numerically higher AUC of 0.712 (95% CI: 0.589–0.825), with overlapping confidence intervals, indicating that detailed FFQ-derived micronutrient data did not improve prediction. Maternal age and pre-pregnancy BMI were the strongest individual predictors by SHAP analysis. No model reached the AUC >0.80 threshold for good discrimination. Substantial miscalibration was observed (slope: 0.56; intercept: −1.83), limiting use for absolute risk estimation. **Conclusions**: This exploratory study demonstrates that first-trimester ML models achieve modest discriminative ability for early GDM prediction, with routine clinical variables performing comparably to models incorporating detailed dietary assessment. These findings should be interpreted with caution, as no external validation cohort was available and the low events-per-variable ratio (~3.8) constrains the reliability of individual model estimates. Substantial miscalibration further limits use for absolute risk estimation. Accordingly, these models should be regarded as exploratory risk-ranking tools only and require external validation and recalibration before any clinical implementation.

## 1. Introduction

Gestational diabetes mellitus (GDM) is one of the most common metabolic complications of pregnancy, affecting approximately 14–15% of pregnancies worldwide, with prevalence continuing to rise in parallel with increasing maternal age and obesity rates [1,2], with outcomes including but not limited to hypertensive disorders of pregnancy (such as preeclampsia), cesarean delivery, fetal macrosomia, neonatal metabolic complications [2,3], and an increased lifetime risk of type 2 diabetes mellitus for both mother and offspring [4,5]. Early identification of women at increased risk for GDM therefore represents an important public health and clinical priority [6].

Current clinical practice relies on universal or risk-based screening using the oral glucose tolerance test (OGTT), typically performed between 24 and 28 weeks of gestation [7]. While this approach is effective for diagnosis, it identifies GDM relatively late in pregnancy, after key metabolic adaptations have already occurred. This timing limits opportunities for early preventive interventions such as targeted nutritional counseling, lifestyle modification [8], or enhanced metabolic monitoring during the first and early second trimesters, during which maternal insulin sensitivity and placental development are rapidly evolving [9]. While earlier testing and improved lab protocols are able to identify high-risk women earlier, early OGTT alone is not sensitive enough to detect all at-risk pregnancies before metabolic deterioration occurs [10,11]. Recent evidence from the TOBOGM randomized controlled trial demonstrated that immediate treatment of GDM diagnosed before 20 weeks’ of gestation led to modestly lower incidence of adverse neonatal outcomes compared to deferred treatment [12], further supporting the clinical value of early GDM identification.

In response to these limitations, increasing attention has been directed towards the development of early prediction models for GDM using data available during early pregnancy or even before conception. Traditional statistical risk scores and regression-based models that combine maternal age, body mass index (BMI), ethnicity, and obstetric history demonstrate modest predictive performance and limited generalizability across populations [13,14]. More recently, machine learning (ML) approaches have been proposed as a means of improving early risk stratification by modeling complex, non-linear relationships [15,16] and interactions among demographic, clinical, lifestyle, and nutritional factors, including dietary intake assessed at early pregnancy and/or reflecting the period before pregnancy initiation. In addition, dietary factors before and during pregnancy have been increasingly recognized as important contributors to maternal glucose metabolism and GDM risk [17]. Pre-pregnancy and early pregnancy dietary patterns, micronutrient and macronutrient intake, and overall diet quality have been associated with altered GDM risk [18,19]. However, dietary assessment in pregnancy is challenging due to physiological and behavioral changes that may affect reporting accuracy [20]. Instruments like food frequency questionnaires (FFQs) rely on memory and are subject to recall bias and difficulties in accurately quantifying portion sizes. Furthermore, estimated dietary intake does not necessarily equate to physiological bioavailability, as individual metabolic differences affect nutrient absorption. Despite these limitations, FFQs remain the standard tool in epidemiological studies to capture habitual intake patterns [21]. While such instruments may not reflect acute dietary changes during early pregnancy, they can provide valuable insight into baseline nutritional status entering pregnancy, which may influence subsequent metabolic adaptation [22]. Moreover, when combined with clinical and demographic data, FFQ data may contribute meaningfully to early risk prediction models, particularly when analyzed using ML approaches capable of handling heterogeneous data with mixed variable types [23].

Consistent with this, multiple studies have reported moderate predictive performance for early GDM models using ML algorithms, with area under the receiver operating characteristic curve (AUC-ROC) values typically ranging from 0.70 to above 0.80 when incorporating early pregnancy clinical data, biochemical markers, or family history of diabetes [24,25,26]. Unlike biochemical markers (such as fasting glucose), which offer a direct readout of metabolic status, dietary intake represents a more distal and ‘noisy’ signal that is harder to capture accurately. In addition, few studies have rigorously examined the impact of class imbalance handling strategies or reported negative findings related to model generalization.

ML approaches offer several potential advantages over traditional statistical methods for clinical prediction tasks: (1) ability to model non-linear relationships, (2) automatic feature interaction detection, (3) flexible handling of heterogeneous predictor types, and (4) systematic comparison of multiple algorithmic approaches. Recent advances in gradient boosting algorithms (such as XGBoost, LightGBM, CatBoost) and neural network architectures have shown promising results in various clinical prediction tasks [27,28].

The present study was designed as an exploratory, development-phase investigation to assess the feasibility and preliminary performance of ML–based prediction of GDM using first-trimester data from a Greek Mediterranean cohort. We specifically aimed to evaluate whether detailed assessment of pre-pregnancy micronutrient intake improves predictive performance compared with routinely collected clinical variables. This work represents an initial attempt to determine whether the complex and potentially ‘noisy’ signal of dietary history can be effectively leveraged by non-linear ML algorithms for early GDM risk stratification.

## 2. Materials and Methods

### 2.1. Study Design and Participants

This study analyzed data from the BORN2020 prospective cohort study, approved by the Ethics Committee of Aristotle University of Thessaloniki (approval number 6.231/29 July 2020). The study was conducted in accordance with the Declaration of Helsinki. All participants provided written informed consent.

Pregnant women were recruited from July 2020 to October 2022 at the 3rd Department of Obstetrics and Gynecology, School of Medicine, Faculty of Health Sciences, Aristotle University of Thessaloniki, Greece. Inclusion criteria were: (1) age ≥ 18 years, (2) singleton pregnancy, (3) no pre-existing diabetes mellitus, and (4) attending first prenatal visit at 11^+0^ to 13^+6^ weeks of gestation. Exclusion criteria included (1) multiple pregnancy, (2) serious medical conditions affecting metabolism, (3) women following special diets (e.g., vegan), and (4) incomplete data for key variables.

### 2.2. Data Collection

**Demographic and anthropometric data:** All data were collected during a single routine first-trimester prenatal visit corresponding to the standard fetal ultrasound examination, conducted at approximately 11^+0^ to 13^+6^ weeks of gestation. Maternal age and height were measured at enrollment. Pre-pregnancy weight was obtained at the first prenatal visit; weight measured at the time of the visit was assumed to approximate pre-pregnancy weight, as minimal weight change is typically expected in early pregnancy [29]. This assumption was applied uniformly across all participants. Pre-pregnancy BMI was calculated as weight (kg) divided by height squared (m^2^) and categorized according to WHO classifications: underweight (<18.5 kg/m^2^), normal weight (18.5–24.9 kg/m^2^), overweight (25.0–29.9 kg/m^2^), and obese (≥30 kg/m^2^) [30].

**Clinical characteristics:** Parity, history of assisted reproductive technology (ART), thyroid disease, and current smoking status were obtained through structured interviews and medical record reviews.

**Lifestyle factors:** Physical activity was assessed using the International Physical Activity Questionnaire Short Form (IPAQ-SF), validated for use in pregnancy. Walking activity (>10 min per day) was recorded as frequency per week.

**Dietary assessment:** Dietary intake was assessed using a validated semi-quantitative Food Frequency Questionnaire (FFQ) that captured habitual dietary intake during the six months preceding pregnancy. The FFQ has been validated for use in Greek population. Although dietary data were collected during the first-trimester visit, the FFQ primarily reflects pre-pregnancy dietary habits rather than dietary intake exclusively during early pregnancy. Dietary micronutrient intakes were calculated using NutriSurvey software (last software update 29 October 2007) and included vitamins (A, B1, B2, B6, B12, C, D, E, K, folate, niacin) and minerals (calcium, iron, zinc, magnesium, phosphorus, potassium, sodium, copper, manganese, iodine).

**Data collection timing:** All predictors were collected at the first-trimester visit; however, some variables (dietary intake and pre-pregnancy weight) were retrospectively assessed. Accordingly, the term “first-trimester data” in this study refers to the timing of data collection rather than the exact exposure period for all predictors.

### 2.3. GDM Diagnosis

GDM was diagnosed using the Hellenic Society of Obstetrics and Gynecology (HSOG) criteria, which are based on the Hyperglycemia and Adverse Pregnancy Outcome (HAPO) study thresholds [31]. All participants underwent a 75 g oral glucose tolerance test (OGTT) at 24–28 weeks of gestation. GDM was diagnosed if any of the following plasma glucose values were met or exceeded: fasting ≥ 92 mg/dL (5.1 mmol/L), 1-h ≥ 180 mg/dL (10.0 mmol/L), or 2-h ≥ 153 mg/dL (8.5 mmol/L).

### 2.4. Feature Selection and Engineering

A total of 31 features were used for model development, selected based on clinical relevance and data availability at the first trimester. The following features were included:

Demographics (4 features): Maternal age (continuous), pre-pregnancy weight (kg), height (cm), BMI (kg/m^2^). Although BMI is mathematically derived from weight and height, all three variables were retained to allow the model to learn non-linear and non-proportional relationships between body size components and GDM risk. L2 regularization applied to Logistic Regression mitigates coefficient instability arising from this collinearity. Clinical characteristics (3 features): ART (binary), thyroid condition (binary), smoking status (binary). Obstetric (1 feature): Parity (continuous count). Lifestyle (1 feature): Walking frequency (times per week). Dietary micronutrients (22 features): Absolute (unadjusted) vitamin and mineral intakes including vitamins (A, B1, B2, B6, B12, C, D, E, K, folic acid as dietary folate equivalents, niacin, retinol) and minerals (calcium, iron, zinc, magnesium, phosphorus, potassium, sodium, copper, manganese, iodine). Equivalent forms (vitamin E equivalents and niacin equivalents) were excluded as they were redundant with their respective base forms already included in the feature set. Folic acid was retained as dietary folate equivalents (DFE) since no base folic acid column was available.

To assess whether detailed dietary micronutrient data provided incremental predictive value beyond standard clinical information, we pre-specified a comparison between the full 31-feature model and a reduced 9-feature model containing only routinely collected clinical and demographic variables (maternal age, pre-pregnancy weight, height, BMI, parity, ART, thyroid condition, smoking status, walking frequency). This comparison addresses a primary research question: whether the resource-intensive FFQ-based dietary assessment improves early GDM prediction beyond variables available at any standard first prenatal visit. Dietary micronutrient variables were included as absolute intake values without energy adjustment (e.g., nutrient density or residual method). It is important to note that this study adopts a predictive modeling framework rather than an etiologic one; consequently, absolute intakes were used to maximize predictive signal and clinical usability rather than to isolate independent nutrient effects. In traditional nutritional epidemiology, energy adjustment (such as the nutrient residual method) is essential to separate specific nutrient effects from overall energy intake. Without such adjustment, absolute micronutrient intakes largely serve as proxies for total energy consumption, and the model may be capturing associations between higher overall dietary intake, correlated metabolic characteristics (e.g., higher BMI), and GDM risk, rather than independent micronutrient effects. As a sensitivity analysis, energy-adjusted micronutrient values were computed using Willett’s nutrient residual method [32], regressing each micronutrient on total energy intake across the full population and using the residuals (shifted by the population mean) as model inputs. This analysis did not improve predictive performance across any model; for the best-performing configuration under energy-adjusted inputs (MLP without resampling), the test AUC was 0.725 versus 0.720 for the same model with unadjusted inputs, supporting the use of absolute intakes in the primary analysis while acknowledging that the observed micronutrient associations likely reflect, at least in part, total energy intake patterns rather than independent nutrient effects. Therefore, SHAP-derived feature importance values for individual micronutrients should be interpreted as reflecting predictive contributions within this specific model—potentially driven by total energy intake patterns—rather than evidence for independent biological effects of specific nutrients on GDM risk. This is an important limitation that should be considered when interpreting the dietary findings of this study.

Regarding multicollinearity, we note that several dietary features are moderately to highly correlated, as expected given overlapping food sources (e.g., calcium and phosphorus are both concentrated in dairy products). However, multicollinearity poses a different concern in ML compared to classical regression. For tree-based ensemble methods (Random Forest, XGBoost, LightGBM), correlated features do not affect prediction accuracy but may dilute individual feature importance, as the model distributes credit across correlated predictors. For Logistic Regression, L2 regularization (ridge penalty) was applied, which stabilizes coefficient estimates in the presence of collinearity without requiring manual feature removal. SHAP values, used for interpretability analysis, account for feature interactions and provide more reliable importance estimates than raw model coefficients in the presence of correlated features. This limitation should be considered when interpreting individual feature contributions. Given the relatively low events-per-variable ratio in the training dataset, SHAP-derived feature importance rankings should be interpreted as exploratory rather than confirmatory. While SHAP provides internally consistent explanations of model behavior, feature rankings may be sensitive to sampling variability in low-event settings and should not be interpreted as stable estimates of causal or independent predictive effects.

Features were named with the suffix ‘-A’ to denote first-trimester timing, ensuring temporal validity of predictions. Missing values (<5% for all features) were imputed using median imputation. Continuous features were standardized using StandardScaler for algorithms requiring scaled inputs (Logistic Regression, Neural Networks).

### 2.5. Machine Learning Algorithms

We evaluated 10 machine learning algorithms spanning different model families: Linear models: Logistic Regression. Tree ensembles: Random Forest, Extra Trees. Gradient boosting: XGBoost, LightGBM, CatBoost, Gradient Boosting, AdaBoost. Meta-ensembles: Bagging Classifier. Neural networks: Multi-Layer Perceptron (MLP). All algorithms were implemented using scikit-learn (version 1.6.0), XGBoost, LightGBM, and CatBoost. CatBoost was tuned using manual cross-validation due to incompatibility with scikit-learn’s GridSearchCV in version 1.6+.

### 2.6. Hyperparameter Optimization

For each algorithm, hyperparameters were optimized using exhaustive grid search with 5-fold stratified cross-validation on the training set. The complete hyperparameter search spaces are provided in Supplementary Table S1. The best hyperparameters were selected based on maximum cross-validation AUC-ROC.

### 2.7. Class Imbalance Handling

Given the class imbalance (GDM prevalence), we evaluated five sampling strategies:

1. None: No resampling, using original class distribution
2. SMOTE: Synthetic Minority Over-sampling Technique
3. Borderline SMOTE: SMOTE variant focusing on borderline cases
4. ADASYN: Adaptive Synthetic Sampling
5. SMOTE-Tomek: Combined over- and under-sampling

All sampling strategies used a target ratio of 0.6 (60% minority class proportion after resampling) and were applied only to the training set, never to the test set.

### 2.8. Model Training and Validation

Data were split into training (80%, *n* = 637) and test (20%, *n* = 160) sets using stratified sampling to preserve GDM prevalence in both sets. Missing values were imputed using the median of the training set, and the same imputed values were applied to the test set to prevent information leakage. Similarly, feature standardization (z-score normalization) was performed using parameters (mean and standard deviation) derived exclusively from the training set, which were then applied to transform the test set. Stratified 5-fold cross-validation was used for hyperparameter tuning on the training set. Final model performance was evaluated on the held-out test set.

Models were trained with a random state fixed at 42 for reproducibility. No post-test model retraining was performed to ensure unbiased performance estimates.

To further characterize model performance, the development dataset was partitioned into a training set and an internal hold-out test set (20% of data, *n* = 160) reserved prior to any model training or tuning. Additionally, bootstrap resampling with 1000 iterations was employed to estimate 95% confidence intervals for AUC-ROC on the test set, providing robust measures of performance uncertainty. The cross-validation to test set performance gap analysis (Section 3.4) further assessed the optimism in model performance estimates.

The effective events-per-variable (EPV) ratio in the training set was approximately 3, with 94 GDM events and 31 candidate predictors. This EPV is below commonly recommended thresholds for reliable estimation in clinical prediction modeling, particularly for flexible machine learning algorithms [33]. Accordingly, model development emphasized strong internal validation, regularization, and conservative interpretation of performance estimates [34].

Model development followed a strict separation of training and evaluation data: (1) a single stratified 80/20 train–test split was performed prior to any model training; (2) hyperparameter tuning and all resampling were performed exclusively within the training set using 5-fold stratified cross-validation; (3) classification thresholds were derived from cross-validation folds (mean Youden’s index = 0.449); (4) final performance was assessed once on the held-out test set using the pre-specified threshold, with no post-test retraining or threshold adjustment. This design ensures that no information from the test set influenced model development at any stage.

Given the low EPV (≈3.8), all model comparisons, coefficient estimates, and SHAP-derived feature importance rankings should be interpreted as exploratory and hypothesis-generating rather than definitive. The low EPV favors simpler, regularized models and increases the risk of instability for flexible ML algorithms, consistent with established guidance for clinical prediction modeling [33,34].

This study employed a development-plus-internal-test-split design. No external validation cohort was available, which represents a key limitation; all reported performance estimates should be considered provisional until validated in an independent population.

### 2.9. Performance Metrics

The primary performance metric was the AUC-ROC. Given the clinical screening context, NPV and threshold-dependent sensitivity/specificity trade-offs were considered co-primary metrics alongside AUC-ROC, as these directly reflect the model’s practical utility for ruling out low-risk pregnancies. Secondary metrics included:

- Sensitivity (Recall): TP/(TP + FN).
- Specificity: TN/(TN + FP).
- Precision (Positive Predictive Value): TP/(TP + FP).
- Negative Predictive Value (NPV): TN/(TN + FN).
- F1 Score: 2 × (Precision × Recall)/(Precision + Recall).
- Accuracy: (TP + TN)/(TP + TN + FP + FN).
- Brier Score: Mean squared difference between predicted probabilities and actual outcomes.

Confidence intervals (95%) for AUC-ROC were calculated using bootstrap resampling with 1000 iterations. To avoid optimistic bias from threshold selection on test data, optimal classification thresholds were determined using Youden’s index (sensitivity + specificity − 1) computed as the average of Youden-optimal thresholds across the five cross-validation folds of the training set (mean threshold = 0.449, SD = 0.025). This pre-specified threshold was then applied to the independent internal hold-out test set. AUC-ROC, being threshold-independent, was not affected by this procedure. Youden’s index was selected as the primary threshold optimization criterion because it maximizes the sum of sensitivity and specificity, providing an unbiased balance between detecting true GDM cases and correctly classifying non-GDM pregnancies. While alternative threshold selection methods exist—including maximizing the F1 score (which favors precision–recall balance), fixing sensitivity at a predefined level (e.g., ≥0.80), and cost-based optimization—Youden’s index is widely used in clinical prediction studies and recommended when no strong prior preference exists for prioritizing sensitivity over specificity or vice versa.

Although Youden’s index was selected as the primary threshold optimization criterion to provide an unbiased balance between sensitivity and specificity simultaneously and provides a balanced, unweighted operating point without requiring prior assumptions about the relative clinical cost of false positives versus false negatives [35], fixed-sensitivity operating points (approximately 80% and 90% sensitivity) were also evaluated to reflect potential screening-oriented use cases in early pregnancy.

Decision curve analysis (DCA) was performed to evaluate the clinical utility of the best-performing model across a range of threshold probabilities [36]. DCA quantifies the net benefit of using the model compared to default strategies of treating all or no patients, accounting for the relative harms of false positives and false negatives at each decision threshold. Bootstrap resampling (500 iterations) was used to estimate 95% confidence intervals for the net benefit curve.

### 2.10. Model Interpretability

To assess the relative contribution of individual features to model predictions, SHAP (SHapley Additive exPlanations) analysis was performed on the best-performing model [37]. SHAP values, derived from cooperative game theory, provide a theoretically grounded, model-agnostic measure of each feature’s contribution to individual predictions, accounting for feature interactions and nonlinearities [37]. For the best-performing Logistic Regression model, SHAP KernelExplainer was used with background data summarized using k-means clustering (k = 50) to approximate Shapley values [37]. While exact SHAP values can be computed for linear models using LinearExplainer, KernelExplainer was selected to maintain methodological consistency across all algorithm comparisons, as the same explainer was applied to non-linear models (gradient boosting, neural networks) where LinearExplainer is not applicable. This choice prioritizes comparability across the full model benchmarking experiment at the cost of introducing approximation error for the Logistic Regression model specifically. As a verification, SHAP LinearExplainer—which computes exact Shapley values for linear models—was applied to the final Logistic Regression model, producing a highly consistent feature ranking (Spearman ρ = 0.982, *p* < 0.001; 5/5 top-5 features identical; Supplementary Table S6 and Supplementary Figure S1). The k-means summarization of background data balances computational efficiency with estimation accuracy [38]. For each participant in the test set, SHAP values quantify the direction and magnitude of each feature’s effect on the predicted GDM probability. Mean absolute SHAP values across all test-set predictions were used as the global feature importance metric. Features were grouped into five categories (demographic, clinical, obstetric, lifestyle, and micronutrient dietary data) to enable category-level comparison of predictive contributions. Both total category importance and per-feature average importance were calculated to account for differences in the number of features across categories.

### 2.11. Statistical Analysis

Baseline characteristics were compared between the GDM and non-GDM groups using appropriate statistical tests. Continuous variables were analyzed using the independent *t*-test or the Mann–Whitney U test for non-normally distributed data. Categorical variables were compared using the chi-square test or Fisher’s exact test when expected cell counts were less than five. Normality was assessed using the Shapiro–Wilk test. Statistical significance was set at a two-sided α level of 0.05. All statistical analyses were performed using Python (version 3.12) with SciPy (version 1.14.1).

### 2.12. Ethical Considerations

The study was approved by the Ethics Committee of Aristotle University of Thessaloniki (approval number 6.231/29 July 2020) and conducted in accordance with the Declaration of Helsinki. All participants provided written informed consent. Data were pseudonymized and stored securely in compliance with the General Data Protection Regulation (GDPR).

### 2.13. Supplementary Materials

The Supplementary Materials include: the complete hyperparameter search spaces for all algorithms (Table S1), the full results of all 50 experiments (Table S2), optimal hyperparameters identified through grid search (Table S3), complete SHAP importance values for all 31 features (Table S4), detailed feature definitions with units and clinical relevance (Table S5), comparison of SHAP feature rankings between KernelExplainer and LinearExplainer for the best-performing Logistic Regression model (Table S6), and side-by-side comparison of SHAP feature importance using both explainer methods (Figure S1). Additional methodological details are provided in Supplementary Text S1.

## 3. Results

### 3.1. Study Population Characteristics

A total of 797 pregnant women were included in the analysis, of whom 680 did not develop GDM (85.3%) and 117 developed GDM (14.7%). Table 1 presents baseline characteristics stratified by GDM status.

BMI: Body Mass Index; thyroid disease: including one of hypothyroidism, Hashimoto’s disease, hyperthyroidism; Nan: not a number.

Table 1 shows the differences between baseline characteristics of non-GDM women compared to GDM women. Women in the GDM group were significantly older, with a mean age approximately two years higher than that of the non-GDM group, and a substantially greater proportion met criteria for advanced maternal age (≥35 years), supporting age as an important risk factor (*p* < 0.001 for both comparisons). Pre-pregnancy body mass index was modestly but significantly higher among women who developed GDM, and obesity was nearly twice as prevalent in this group, consistent with the observed difference in median BMI (*p* = 0.004) and higher mean pre-pregnancy weight (*p* = 0.001). In contrast, maternal height did not differ between groups, indicating that differences in BMI were driven primarily by weight rather than stature. Smoking during early pregnancy was also more common among women who developed GDM (*p* = 0.004), whereas no significant differences were observed for parity, use of assisted reproductive technology (ART), thyroid conditions, or walking frequency.

Key findings from baseline comparisons:

- Women who developed GDM were significantly older than those who did not (mean difference ~2 years, *p* < 0.001). Pre-pregnancy BMI was higher in the GDM group (median difference ~1 kg/m^2^, *p* < 0.05). Obesity prevalence was nearly double in the GDM group (21.4% vs. 11.0%). Current smoking was more prevalent in the GDM group (17.9% vs. 8.8%, *p* < 0.01). No significant differences were observed for parity, ART use, or thyroid conditions

### 3.2. Model Performance

**Figure 1:**
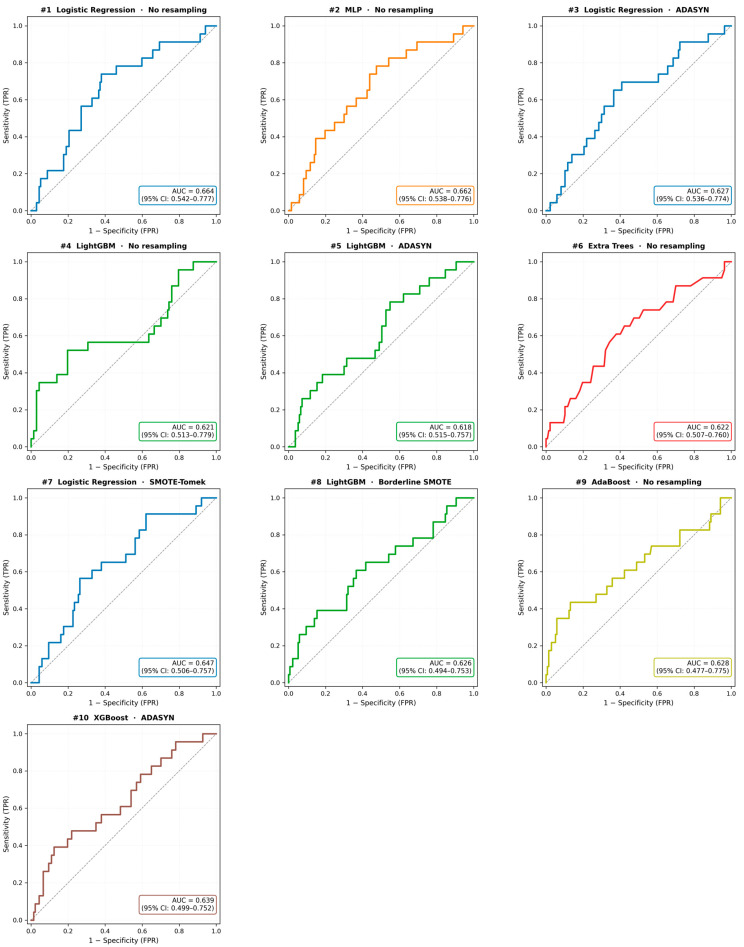
Receiver Operating Characteristic (ROC) curves for the top 10 performing model configurations. Each panel displays the ROC curve for one model–sampling strategy combination, ranked by test set AUC-ROC (rank indicated in panel title). The dashed diagonal line represents random chance (AUC = 0.50). AUC values with 95% bootstrap confidence intervals (1000 iterations) are displayed within each panel.

Among the 50 model configurations tested (10 algorithms × 5 sampling strategies), the top 10 models achieved AUC-ROC values ranging from 0.627 to 0.664. ROC curves for all top 10 configurations are shown in Figure 1. Table 2 presents detailed performance metrics for these models.

AUC-ROC: Area under the receiver operating characteristic curve. CI: Confidence interval. Confidence intervals (95%) were calculated using bootstrap resampling with 1000 iterations. Sensitivity (recall) = TP/(TP + FN); Specificity = TN/(TN + FP); Precision (PPV) = TP/(TP + FP); NPV = TN/(TN + FN); F1 Score = 2 × (Precision × Recall)/(Precision + Recall); Accuracy = (TP + TN)/(TP + TN + FP + FN). Brier score represents the mean squared difference between predicted probabilities and actual outcomes (lower is better). TP: true positive; TN: true negative; FP: false positive; FN: false negative; PPV: positive predictive value; NPV: negative predictive value. All models were evaluated on an independent held-out test set (*n* = 160, 20% of total data) using stratified sampling to preserve GDM prevalence.

The Logistic Regression model without resampling achieved the highest overall discriminative performance, with an AUC of 0.664 (95% CI: 0.542–0.777). At the pre-specified cross-validation-derived threshold (0.449), sensitivity was 0.783 (95% CI: 0.588–0.941) and specificity was 0.496 (95% CI: 0.413–0.582), correctly identifying approximately 78% of GDM cases. Positive predictive value was 0.207 (95% CI: 0.122–0.307) and negative predictive value was 0.932 (95% CI: 0.867–0.986). While this indicates that 93.4% of women predicted as non-GDM were correctly classified, the high NPV is partly attributable to the moderate baseline GDM prevalence (14.7%), which ensures that most women in any sample are true non-GDM cases. The Brier score was 0.250 (95% CI: 0.221–0.278). For context, a non-informative model predicting the marginal prevalence (0.144) for all individuals achieves a Brier score of approximately 0.123, representing the best-calibrated constant predictor, which provides zero discrimination. The elevated Brier score of the prediction model reflects its substantial miscalibration: the calibration slope of 0.56 indicates overconfident predictions, while the large negative intercept (−1.83) indicates systematic underestimation of predicted GDM risk. The Brier score jointly penalizes discrimination and calibration errors; consequently, a model that attempts to discriminate but is miscalibrated can achieve a higher (worse) Brier score than a trivial predictor that makes no attempt at risk stratification. This does not indicate inferior clinical value—the model’s discriminative ability (AUC = 0.664) confirms its capacity to rank individuals by risk—but rather underscores the need for probability recalibration (e.g., Platt scaling or isotonic regression) before predicted probabilities can be used for individual clinical decision-making.

**Figure 2:**
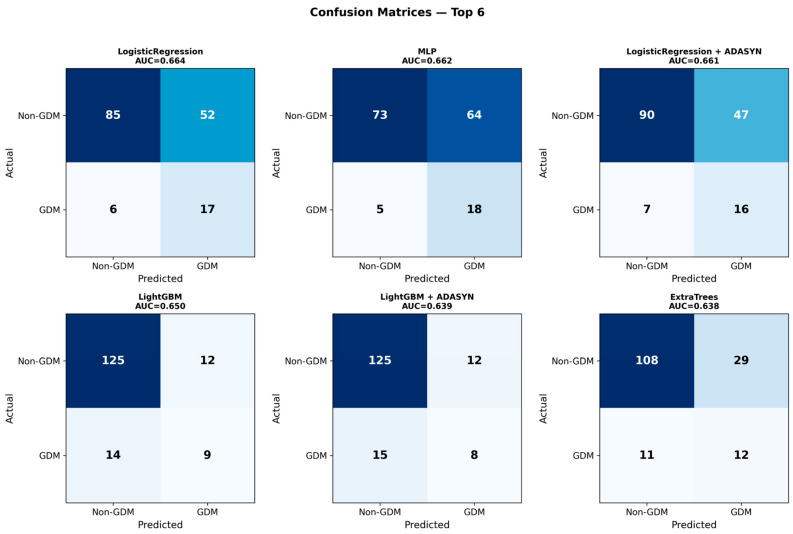
Confusion matrices for the top 6 performing models showing true positives (TP), true negatives (TN), false positives (FP), and false negatives (FN). Test set: *n* = 160 (137 non-GDM, 23 GDM). Confusion matrices visualize classification performance for the top 6 models ranked by AUC-ROC. Each matrix displays the distribution of predictions versus actual outcomes on the independent test set (*n* = 160: 137 non-GDM, 23 GDM cases). Rows represent actual class labels; columns represent predicted class labels. TP (True Positive): Correctly predicted GDM cases; TN (True Negative): Correctly predicted non-GDM cases; FP (False Positive): Non-GDM cases incorrectly predicted as GDM (Type I error); FN (False Negative): GDM cases incorrectly predicted as non-GDM (Type II error). Color intensity reflects the number of cases in each category, with darker shades indicating higher counts. Sensitivity = TP/(TP + FN); Specificity = TN/(TN + FP). Optimal classification thresholds were determined using Youden’s index (sensitivity + specificity − 1). The diagonal from top-left to bottom-right represents correct predictions; off-diagonal elements represent misclassifications.

The MLP (Multi-Layer Perceptron) achieved the second-highest AUC (0.662) with the highest sensitivity among all models (0.783) but lower specificity (0.533). LightGBM without resampling achieved the highest specificity (0.912) among the top 10 models, although with substantially lower sensitivity (0.391). The classification performance of the top 6 models is further illustrated through confusion matrices in Figure 2.

**Figure 3:**
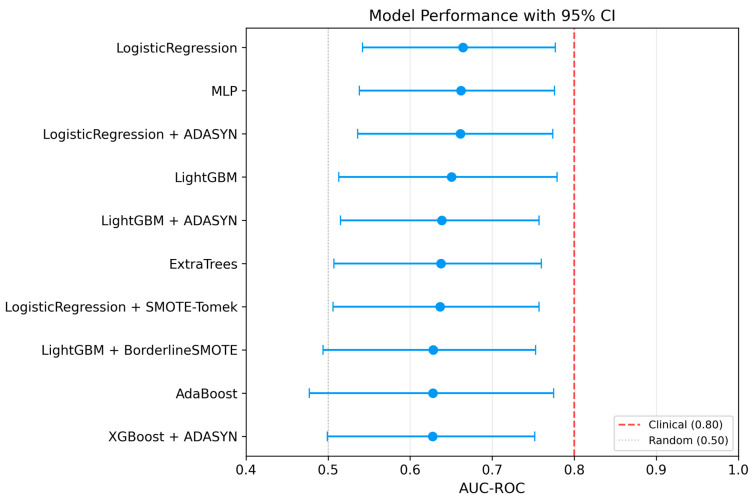
Forest plot showing AUC-ROC point estimates with 95% confidence intervals for the top 10 models. Point estimates are shown as circles with horizontal lines representing 95% bootstrap confidence intervals (1000 iterations). The dashed vertical line indicates the predefined clinical utility threshold (AUC = 0.80). Models are ranked by descending test performance. All metrics were evaluated on an independent held-out test set (*n* = 160).

Despite these findings, none of the evaluated models reached an AUC of 0.80, which is often interpreted as good discriminative ability, though clinical utility ultimately depends on the specific use case, prevalence, and the decision threshold applied (Figure 3).

### 3.3. Impact of Sampling Strategies

Table 3 presents the comparative performance of different sampling strategies for handling class imbalance.

**Figure 4:**
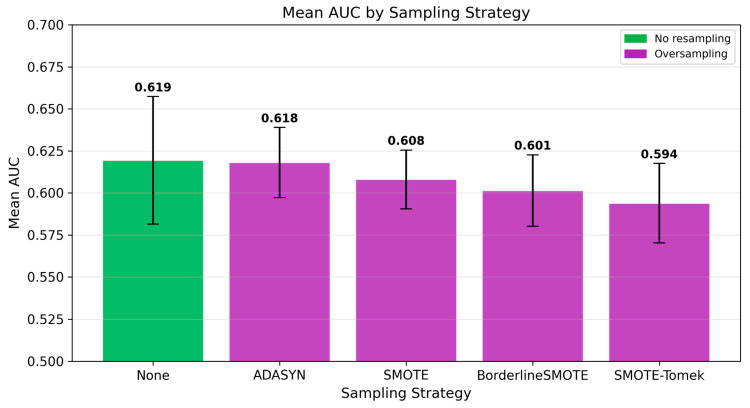
Mean AUC-ROC (±standard deviation) by sampling strategy across all 10 algorithms. Error bars represent standard deviation. Bar plot shows mean AUC-ROC ± standard deviation across all 10 machine learning algorithms for each sampling strategy. Error bars represent standard deviation of performance across algorithms. Sample size (n) indicates the number of algorithm–sampler combinations tested. Green bar highlights the baseline (no resampling) condition; purple bars represent different over-sampling and combined sampling approaches. SMOTE: Synthetic Minority Over-sampling Technique; ADASYN: Adaptive Synthetic Sampling; SMOTE-Tomek: combined over- and under-sampling. All strategies were evaluated on the same independent test set.

The baseline condition (no resampling) achieved the highest mean AUC (0.619) across all algorithms, followed closely by ADASYN (0.618), while SMOTE-Tomek demonstrated the lowest mean AUC (0.594), as illustrated in Figure 4. The absolute difference in performance between the best- and worst-performing sampling strategies was modest (0.025), indicating limited overall impact on model discrimination. Notably, the no-resampling condition matched or outperformed all oversampling strategies, both in mean performance and in achieving the highest individual model AUC (0.664, Logistic Regression). Overall, oversampling techniques, including SMOTE, Borderline-SMOTE, ADASYN, and SMOTE-Tomek, did not consistently improve predictive performance. In several cases, the introduction of synthetic minority samples was associated with reduced model accuracy and increased overfitting (see Section 3.4), suggesting limited benefit of these approaches in the present dataset.

Several models combining gradient boosting algorithms with oversampling techniques exhibited the largest CV–test gaps: LightGBM + ADASYN (test AUC 0.639, gap = 0.297), LightGBM + Borderline SMOTE (gap = 0.313), and XGBoost + ADASYN (gap = 0.311). A likely explanation for this pattern is that oversampling techniques such as SMOTE generate synthetic minority class samples by interpolating between existing instances. In a small dataset (*n* = 637 training samples, 94 GDM cases), these synthetic samples may introduce artificial patterns that high-capacity tree-based models learn to exploit during cross-validation but that do not generalize to the independent test set. Although SMOTE was applied within the cross-validation pipeline (i.e., only to training folds, not before splitting), the limited number of original minority cases may have constrained the diversity of synthetic samples, amplifying this effect. In contrast, models trained on the original class distribution (no resampling) showed minimal CV–test gaps (<5%), suggesting that oversampling was a primary contributor to overfitting in this dataset.

### 3.4. Model Generalization Analysis

To explicitly assess overfitting, cross-validation performance was compared with independent test set performance for all top models, quantifying optimism via CV–test AUC gaps (Table 4). Table 4 compares cross-validation performance with test set performance to assess model generalization and overfitting.

Models without resampling showed excellent generalization, with cross-validation to test set gaps of less than 5% and, in several cases, test performance exceeding cross-validation performance (negative gaps), indicating no overfitting. Logistic Regression and MLP without resampling demonstrated the most stable performance across cross-validation and test sets (gaps of −0.018 and −0.011, respectively). Severe overfitting was observed in three models combining gradient boosting algorithms with oversampling: LightGBM + ADASYN (CV AUC 0.936, test AUC 0.639, gap = 0.297), LightGBM + Borderline SMOTE (gap = 0.313), and XGBoost + ADASYN (gap = 0.311). These results confirm that oversampling techniques induced artificial inflation of cross-validation performance without improving generalization, and their use should be carefully evaluated in clinical prediction studies with moderate sample sizes.

### 3.5. Algorithm-Specific Performance

Table 5 presents the best-performing configuration for each of the 10 machine learning algorithms tested.

Notably, the best-performing configuration for six of the ten algorithms (Logistic Regression, MLP, LightGBM, Extra Trees, AdaBoost, and CatBoost) used no resampling, further supporting the finding that oversampling did not provide a consistent advantage in this dataset. Linear models (Logistic Regression) and neural networks (MLP) outperformed all gradient boosting methods and tree-based ensembles. Given the low events-per-variable ratio (≈3.8) and extensive model selection across multiple algorithms and hyperparameter configurations, comparisons between algorithms should be interpreted as exploratory rather than definitive indicators of model superiority.

### 3.6. Feature Importance Analysis

To assess the relative contribution of individual predictors to model performance, SHAP (SHapley Additive exPlanations) analysis was performed on the best-performing Logistic Regression model (AUC = 0.664). SHAP values quantify each feature’s contribution to individual predictions, providing a unified measure of feature importance. Table 6 presents the top 15 features ranked by mean absolute SHAP values across all test set predictions.

Maternal age (MA) emerged as the single most important predictor, contributing 18.6% of total model importance (mean |SHAP| = 0.090), approximately twice the contribution of the next-ranked feature. The remaining top five features were calcium (9.3%), niacin (8.0%), vitamin B6 (7.7%), and folic acid as dietary folate equivalents (7.5%). Pre-pregnancy BMI ranked sixth (6.7%). Notably, four of the top six predictors were dietary micronutrients, indicating that these features contributed to the model’s predictive performance. However, because micronutrients were included as absolute, non–energy-adjusted intakes, this predictive contribution may reflect associations with total energy intake or overall dietary patterns rather than independent micronutrient-specific effects. The apparent dominance of dietary micronutrients at the aggregate level also reflects the substantially larger number of micronutrient features included (*n* = 22), rather than stronger per-feature predictive power. When normalized per feature, demographic variables demonstrated more than double the average importance of individual micronutrients.

Importantly, the ranking of individual micronutrients should not be interpreted as evidence of nutrient-specific biological or causal effects on GDM risk. Given the use of absolute, non–energy-adjusted intake values and the high collinearity among dietary variables, these micronutrient features likely function as correlated proxies for overall dietary intake patterns or total energy consumption rather than independent mechanistic contributors. Accordingly, SHAP-derived importance values reflect predictive utility within this specific model context and should not be extrapolated to etiologic inference. Importantly, despite their apparent prominence in SHAP rankings, micronutrient features did not improve overall model discrimination in comparative analyses, reinforcing that their predictive importance should not be interpreted as clinically meaningful incremental value.

At the category level, the 22 dietary micronutrient features collectively accounted for 64.1% of total predictive importance, while the four demographic features contributed 26.2%, and clinical (6.1%), obstetric (3.4%), and lifestyle (0.1%) features accounted for the remaining 9.6% (Table 7). However, this apparent micronutrient dominance largely reflects the substantially larger number of dietary features (22 vs. 4) rather than greater individual predictive power. When examining per-feature importance, demographic features averaged 6.6% per feature compared to 2.9% per feature for micronutrients—indicating that individual demographic predictors carried more than double the weight of individual micronutrients.

**Figure 5:**
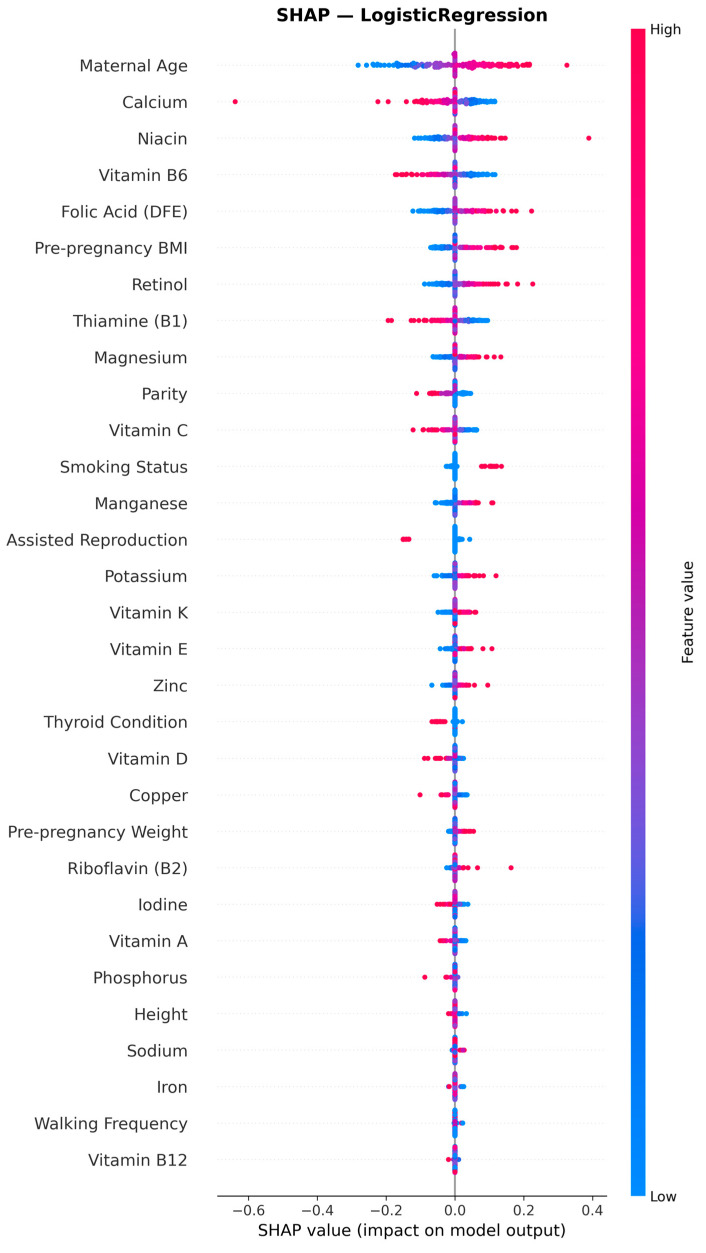
SHAP (SHapley Additive exPlanations) beeswarm plot showing the contribution of each feature to Logistic Regression model predictions. Each dot represents one participant in the test set (*n* = 160). Horizontal position indicates the SHAP value: positive values (right) increase predicted GDM probability, negative values (left) decrease it. Color represents the original feature value (red = high, blue = low). Features are ranked by mean absolute SHAP value from top to bottom. Note: The apparent dominance of dietary micronutrient features in this plot reflects their higher count (22 micronutrient features vs. 4 demographic features) rather than stronger per-feature predictive contribution; on a per-feature basis, demographic variables demonstrated more than double the average importance of individual micronutrients (6.6% vs. 2.9% per feature). See Table 7 for category-level per-feature average importance.

Among the micronutrient predictors, the five most influential were: calcium (9.3%), niacin (8.0%), Vitamin B6 (7.7%), folic acid (7.5%), and retinol (6.1%). These five micronutrients collectively accounted for approximately 38.6% of total model importance. The SHAP beeswarm plot (Figure 5) revealed interpretable directional patterns for several features. As expected, higher maternal age and higher pre-pregnancy BMI were associated with increased predicted GDM probability (positive SHAP values). The linear nature of the Logistic Regression model ensures that SHAP values directly reflect each feature’s additive contribution to the predicted log-odds of GDM, facilitating clinical interpretation. Among micronutrients, the directional patterns observed suggest complex relationships between pre-pregnancy dietary intake and GDM risk that warrant further investigation in prospective studies.

The complete results of all 50 experiments (10 algorithms × 5 sampling strategies) are presented in Supplementary Table S1. The hyperparameter search spaces explored for each algorithm are detailed in Supplementary Table S2, while the optimal hyperparameters identified through grid search with 5-fold cross-validation are reported in Supplementary Table S3. Complete SHAP importance values for all 31 features are available in Supplementary Table S4. Detailed feature definitions, including units, variable types, and clinical relevance to GDM, are provided in Supplementary Table S5. The rationale for feature selection, including justification for included and excluded variable categories, is presented in Supplementary Text S1.

### 3.7. Threshold Analysis for Clinical Application

**Figure 6:**
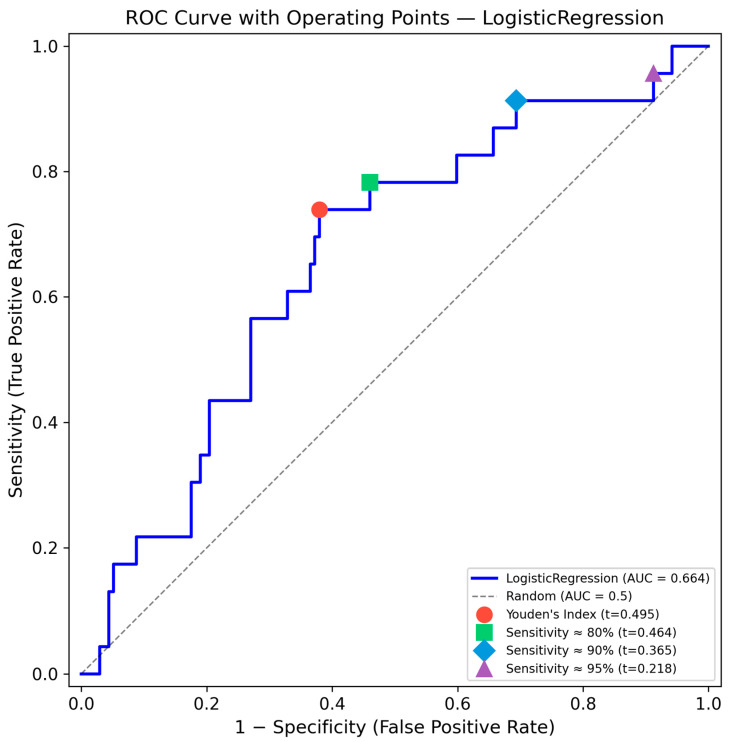
ROC curve of the best-performing Logistic Regression model with operating points at Youden’s index, 80%, and 90% sensitivity thresholds. Point estimates and threshold values are indicated for each operating point.

To evaluate clinical applicability at higher sensitivity levels, the best-performing Logistic Regression model was assessed at fixed sensitivity thresholds (Table 8 and Figure 6). At the cross-validation-derived Youden threshold (0.449), sensitivity was 0.783 (95% CI: 0.588–0.941) with specificity 0.496 (95% CI: 0.413–0.582). At approximately 80% sensitivity (threshold = 0.464), specificity decreased to 0.540 (95% CI: 0.457–0.628), with PPV = 0.222 (95% CI: 0.132–0.325) and NPV = 0.937 (95% CI: 0.878–0.987). At approximately 90% sensitivity (threshold = 0.365), specificity was 0.307 (95% CI: 0.231–0.387), with PPV = 0.181 (95% CI: 0.110–0.254) and NPV = 0.955 (95% CI: 0.879–1.000). These results demonstrate the inherent trade-off in clinical screening: increasing sensitivity to capture more GDM cases reduces specificity and PPV, while NPV remains consistently high (≥0.93), suggesting limited potential for identifying a subgroup of lower-risk women, although the proportion of women classified as low risk remains modest due to low specificity at high sensitivity thresholds. At the 90% sensitivity operating point, specificity was approximately 30%, meaning that nearly 70% of women would still be classified as high risk and therefore undergo standard OGTT screening, limiting any meaningful reduction in testing burden compared with universal screening.

Youden’s index threshold was pre-specified as the average Youden-optimal threshold across 5-fold cross-validation on the training set (0.449), then applied to the internal hold-out test set.

### 3.8. Incremental Value of Dietary Micronutrient Features

The reduced 9-variable model was evaluated to test whether routinely available clinical variables alone could achieve comparable discrimination, thereby directly assessing the incremental predictive value of FFQ-derived micronutrient data. Specifically, we compared the full model (31 features including 22 dietary micronutrients) with a reduced model using only demographic and clinical features (maternal age, pre-pregnancy weight, height, pre-pregnancy BMI, ART, thyroid condition, smoking status, parity, and walking frequency; 9 features). The best-performing basic-only model (MLP) achieved an AUC of 0.712 (95% CI: 0.589–0.825) with sensitivity 0.696 and specificity 0.723, compared to the best full model (Logistic Regression, AUC = 0.664, 95% CI: 0.542–0.777). The basic-only Logistic Regression achieved an AUC of 0.679 (95% CI: 0.538–0.804). The basic-only MLP achieved a numerically higher AUC than the best full model, and the basic-only Logistic Regression also outperformed its full-model counterpart (0.679 vs. 0.664). Although overlapping confidence intervals preclude definitive conclusions about statistical superiority, these results consistently indicate that the addition of 22 dietary micronutrient features did not improve—and may have slightly degraded—predictive performance. Taken together, these findings indicate that the inclusion of detailed FFQ-derived micronutrient data did not improve early GDM prediction beyond routinely collected demographic and clinical variables and, in some configurations, may have introduced additional noise, particularly in a low-event setting.

### 3.9. Calibration Analysis

**Figure 7:**
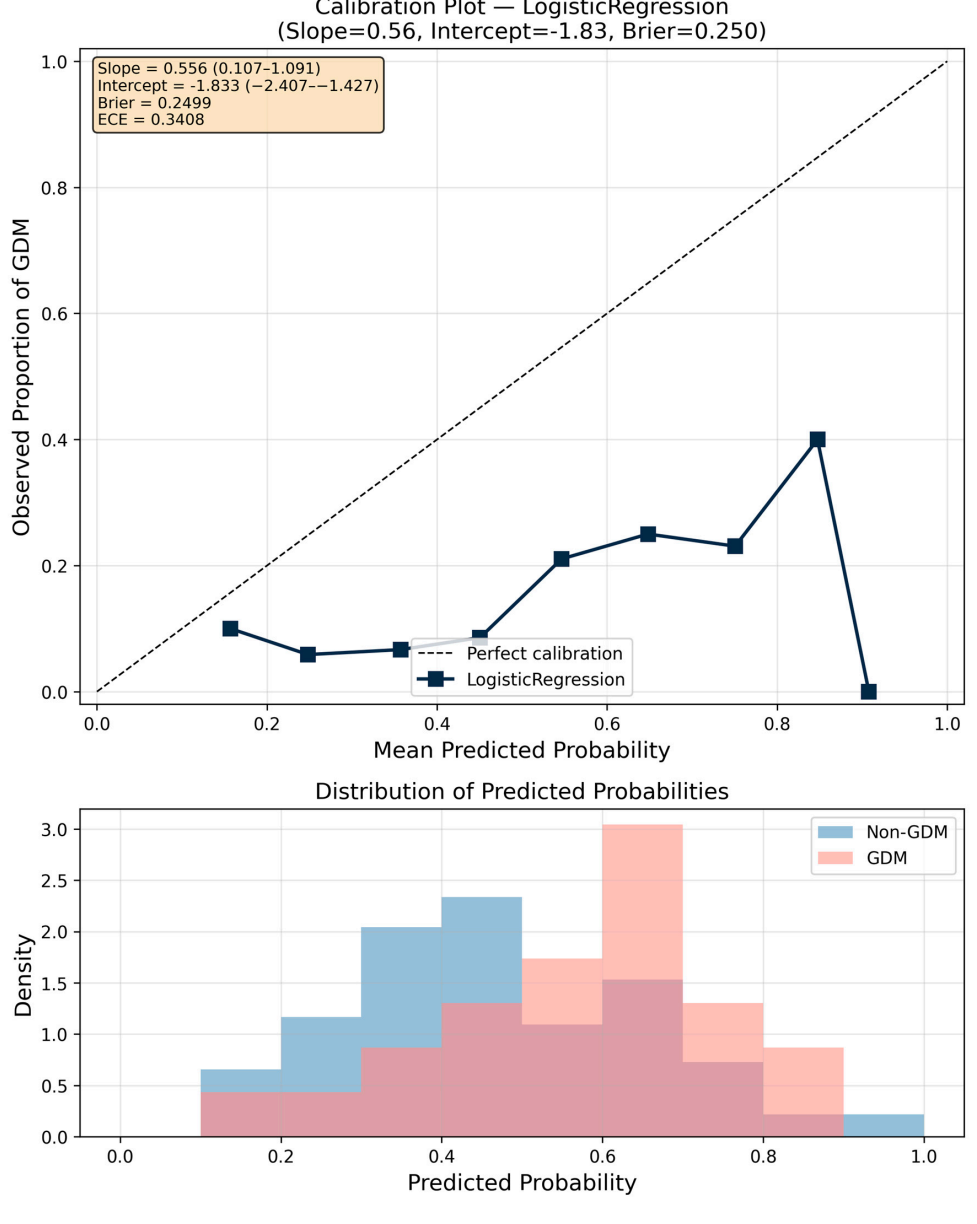
Calibration reliability diagram for the best-performing Logistic Regression model. The dashed diagonal line represents perfect calibration. Calibration slope = 0.56 (95% CI: 0.11–1.09), intercept = −1.83 (95% CI: −2.41 to −1.43). Brier score = 0.250. Confidence intervals for individual calibration points are not displayed on the plot; 95% bootstrap confidence intervals for the calibration slope and intercept are reported in the main text.

Figure 7 presents the calibration plot for the best-performing Logistic Regression model. The calibration slope was 0.56 (95% CI: 0.11–1.09; ideal = 1.0) and the calibration intercept was −1.83 (95% CI: −2.41 to −1.43; ideal = 0.0). The mean predicted GDM probability in the test set was substantially lower than the observed prevalence (14.7%), confirming poor calibration-in-the-large and consistent underestimation of absolute risk. The calibration intercept of −1.83 indicates systematic underestimation of absolute GDM risk, while the calibration slope of 0.56 indicates overly extreme predicted probabilities (i.e., overconfident predictions). Together, these findings demonstrate that although the model preserves modest discriminative ability, its predicted probabilities are not reliable for direct clinical risk estimation without recalibration. These findings represent substantive miscalibration that would require correction before clinical deployment. Although the wide confidence intervals (particularly for the slope, which encompasses 1.0) reflect in part the limited test set size (*n* = 160, 23 GDM cases), the direction and magnitude of miscalibration are clinically meaningful and should not be attributed solely to sampling variability. The Brier score was 0.250 (95% CI: 0.221–0.278), and the expected calibration error (ECE) was 0.341. Formal probability recalibration (e.g., logistic recalibration or isotonic regression) was not performed in this study, as the primary objective was comparative model development rather than deployment; however, future work should evaluate recalibrated models in an independent cohort to assess absolute risk usability. Accordingly, predicted probabilities generated by the current models should not be used for individual-level clinical decision-making, counseling, or risk communication without formal recalibration in an independent population.

### 3.10. Decision Curve Analysis

**Figure 8:**
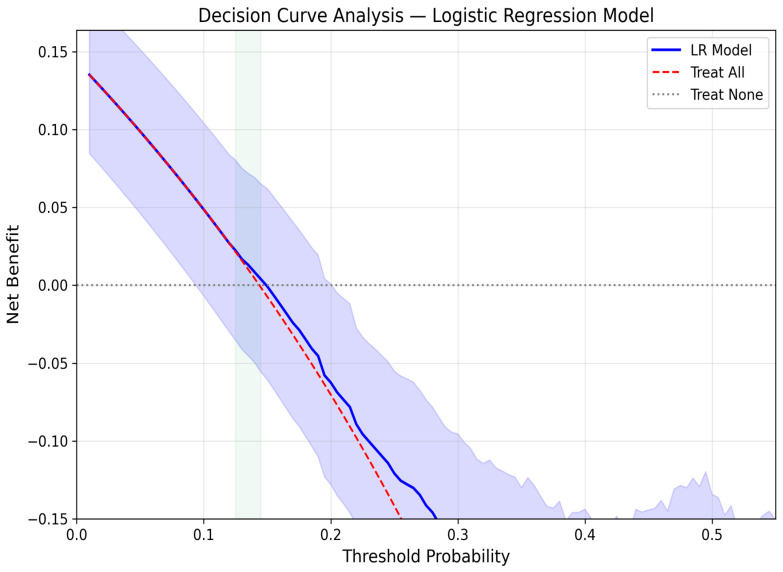
Decision curve analysis for the best-performing Logistic Regression model (AUC = 0.664). Net benefit is plotted against threshold probability for the model (blue line; blue shaded area represents the 95% confidence interval derived from 500 bootstrap iterations), ‘treat all’ strategy (red dashed), and ‘treat none’ strategy (gray dotted). The green shaded area highlights the threshold range over which the model provides positive net benefit compared to the ‘treat all’ strategy (approximately 12.5–14.5%). The model provides positive net benefit over ‘treat all‘ across this narrow range surrounding the observed GDM prevalence (14.7%).

Decision curve analysis (Figure 8) [36] evaluated the clinical utility of the best-performing Logistic Regression model across a range of threshold probabilities. The model provided positive net benefit compared to the default strategies of ‘treat all’ and ‘treat none’ across threshold probabilities from approximately 12.5% to 14.5%, a narrow range surrounding the observed GDM prevalence (14.4%). At thresholds beyond 15%, the model continued to provide marginal net benefit over the ‘treat all’ strategy, though both strategies yielded negative absolute net benefit, reflecting the challenge of screening for a condition with moderate prevalence using a model with modest discriminative ability. These findings indicate that the model’s primary clinical value lies in the threshold range near the population prevalence, where it can marginally improve upon the default strategy of universal screening. The narrow threshold range over which positive net benefit was observed suggests that any potential clinical utility would be limited and context-specific, reinforcing the exploratory nature of the present models.

## 4. Discussion

### 4.1. Main Findings

The present study developed ML models for early prediction of gestational diabetes mellitus using data collected at the first-trimester visit from the BORN2020 Greek cohort. Dietary variables reflected habitual intake before pregnancy (for up to six months prior) and were included as baseline nutritional predictors rather than acute first-trimester exposures. To our knowledge, this represents one of the first attempts to develop ML-based GDM prediction models specifically for a Greek Mediterranean population, incorporating comprehensive dietary assessment data from a locally validated FFQ.

The main findings of this study can be summarized as follows: (1) the best-performing model (Logistic Regression without resampling) demonstrated moderate discriminative ability for early GDM prediction at 11^+0^–13^+6^ weeks of gestation (AUC 0.664, 95% CI 0.542–0.777), with sensitivity of 0.783 and specificity of 0.496 at a cross-validation-derived classification threshold, and none of the evaluated models reached the predefined clinical utility threshold of AUC > 0.80, a commonly cited benchmark for good discrimination in clinical prediction models; (2) critically, a reduced model using only nine basic clinical features (without dietary micronutrients) achieved comparable or numerically superior discriminative performance (AUC 0.712, 95% CI: 0.589–0.825 for MLP; AUC 0.679 for Logistic Regression), indicating that unadjusted dietary micronutrient features did not improve prediction and may have introduced noise or redundancy, suggesting that routinely collected clinical data alone may be sufficient for early GDM risk stratification; (3) Logistic Regression achieved both the highest AUC and a high sensitivity (0.739), correctly identifying approximately three-quarters of GDM cases, while offering the best balance of performance, interpretability, and stability; MLP achieved comparable AUC (0.662) with even higher sensitivity (0.783) but at the cost of lower specificity (0.533); (4) maternal age emerged as the dominant predictor, contributing 18.6% of total SHAP importance, followed by pre-pregnancy BMI (6.7%); collectively, the four demographic features accounted for 26.2% of predictive importance, while the 22 micronutrient features contributed 64.1% at the aggregate level; however, this contribution was driven by feature count rather than per-feature predictive strength and did not translate into improved discriminative performance; (5) oversampling techniques (SMOTE, Borderline SMOTE, ADASYN, SMOTE-Tomek) did not consistently improve model performance, and in several cases led to severe overfitting, with cross-validation to test set AUC gaps exceeding 0.29 for gradient boosting methods combined with oversampling; (6) models without resampling demonstrated excellent generalization with minimal cross-validation to test set performance gaps (<5%), whereas models combining gradient boosting algorithms with oversampling exhibited severe overfitting; (7) the consistently high negative predictive value (≥0.89 across all top models, reaching 0.932 for the best model) largely reflects the moderate baseline prevalence (14.7%) and suggests limited potential for identifying a lower-risk subgroup rather than a robust rule-out strategy; and (8) calibration analysis revealed substantive miscalibration (calibration intercept = −1.83 indicating systematic underestimation of absolute risk; slope = 0.56 indicating overly dispersed predictions), indicating that the model requires recalibration before any clinical application.

Additionally, given the limited number of outcome events, SHAP feature rankings should be interpreted as sample-dependent and hypothesis-generating rather than stable estimates of predictor importance.

Several additional methodological considerations warrant discussion. First, the low EPV increases variance in model estimates and limits the stability of feature rankings, favoring simpler regularized models and conservative interpretation of all performance metrics. Second, model stability was assessed using both CV–test performance gaps and bootstrap confidence intervals: simpler models exhibited narrow uncertainty and minimal optimism, whereas severe optimism was confined to oversampled gradient-boosting models, providing direct evidence that overfitting was model- and strategy-specific rather than pervasive. Third, the reported test set performance reflects the best-performing configuration among 50 tested combinations. Although no post-test retraining was performed, the act of selecting the best model among multiple candidates introduces a degree of selection optimism that cannot be fully quantified without external validation. The narrow spread of AUC values across the top 10 models (0.627–0.664, range = 0.037) partially mitigates this concern, but the best AUC of 0.664 should nonetheless be interpreted as a potentially optimistic estimate of true discriminative ability.

Notably, the finding that the best-performing algorithm was Logistic Regression—an inherently linear model—rather than any of the non-linear approaches (gradient boosting, neural networks) is consistent with the low EPV ratio and moderate sample size. In settings with limited events, simpler models with fewer effective parameters tend to generalize better than flexible non-linear approaches, as the latter may overfit to noise in the training data. This finding aligns with recommendations from clinical prediction modeling literature favoring parsimony when sample sizes are limited.

The better generalization of Logistic Regression compared with more complex machine learning algorithms is likely explained by the limited effective sample size and low events-per-variable ratio. Methodological guidance for clinical prediction modeling indicates that low EPV settings favor simpler, regularized models and substantially increase the risk of overfitting for flexible machine learning approaches, particularly when extensive hyperparameter tuning and model selection are performed [34]. This is consistent with the severe optimism observed for gradient boosting models combined with oversampling in the present study, contrasted with the stability of Logistic Regression and MLP models trained without resampling.

A key limitation of this study is the substantial miscalibration of predicted probabilities. Despite modest discrimination, calibration analysis revealed a markedly reduced slope (0.56) and a strongly negative intercept (−1.83), indicating both overconfident predictions and systematic underestimation of absolute GDM risk. The observed miscalibration precludes use of predicted probabilities for individual risk counseling or clinical decision-making without formal recalibration in an independent cohort. Pending such recalibration and external validation, the model’s potential utility is restricted to relative risk ranking rather than absolute risk estimation.

Overall, these findings suggest that, in moderate-sized obstetric cohorts with limited outcome events, parsimonious models based on routinely available clinical variables may offer comparable or superior performance to high-dimensional feature sets without compromising generalizability. The present results underscore the importance of restraint in feature expansion and the need for external validation before translation of machine learning models into early pregnancy screening pathways. Rather than indicating the superiority of a specific algorithm, the comparable performance of the reduced 9-feature model serves as evidence that high-dimensional dietary features did not add predictive value in this low-EPV setting, reinforcing the principle of parsimony. From a practical standpoint, this suggests that early GDM risk stratification may not require detailed dietary assessment when standard demographic and clinical variables provide equivalent predictive value. Future development efforts may therefore be better directed toward optimizing parsimonious models incorporating biochemical markers (e.g., fasting glucose, HbA1c) rather than expanding dietary feature sets.

### 4.2. Comparison with Previous Literature

Our findings align with several recent studies employing ML approaches for GDM prediction. Wu et al. [15], in a study of a Chinese population comprising over 17,000 pregnant women, achieved an AUC of 0.80 using gradient boosting decision trees with a comprehensive feature set including fasting plasma glucose, triglycerides, and family history of diabetes. Notably, their model benefited substantially from biochemical markers collected in early pregnancy, and when restricting their analysis to clinical features alone, their model’s performance decreased to approximately 0.68, which is comparable to our findings using demographic and lifestyle variables. This observation highlights that the inclusion of metabolic biomarkers substantially enhances predictive accuracy and has important implications for resource-limited settings where extensive laboratory testing may not be feasible during routine first-trimester care.

Another study, conducted by Xiong et al. [16], developed ML models for GDM prediction within the first 19 weeks of pregnancy, reporting an AUC of 0.77 using Random Forest with features including maternal age, BMI, fasting blood glucose, and obstetric history, such as parity and history of previous GDM or macrosomia. Their analysis emphasized the importance of pre-pregnancy BMI as a dominant predictor, which is consistent with our finding that BMI ranked among the top features across all model configurations (6.7% of SHAP-derived importance in our study). In their analysis, BMI contributed 20–35% of predictive importance, likely reflecting its greater relative weight in a feature set without dietary variables.

The inclusion of fasting glucose measurements in their study, a direct marker of glucose metabolism, likely contributed to the performance difference observed compared to models relying exclusively on demographic, lifestyle, and nutritional features, as in the present analysis.

Moreover, Kumar et al. [25] conducted a population-centric study in Singapore, developing machine learning models using XGBoost with demographic and clinical predictors. Their study highlighted the importance of ethnicity-specific risk factors and the value of population-tailored prediction models. Interestingly, their best-performing model utilized a relatively modest feature set of 12 variables, comparable to our basic configuration. The differences in performance across populations emphasize the importance of developing and validating prediction models within specific demographic contexts, as risk factor profiles and their relative contributions may vary substantially across different ethnic groups and healthcare settings.

Cubillos et al. [24] reported findings from a Chilean cohort using ML for first-half pregnancy GDM prediction, achieving an AUC of 0.74 with gradient boosting methods, a result closely aligned with our findings. Their results are particularly relevant to our study as they similarly examined a population with comparable GDM prevalence rates. Importantly, they observed significant overfitting with ensemble methods, consistent with our experience where tree-based models showed substantial gaps between cross-validation and test set performance. This phenomenon appears to be characteristic of obstetric cohort studies with moderate sample sizes, suggesting that regularized models and rigorous validation strategies should be prioritized in this context.

Two recent publications based on the same BORN2020 cohort provide complementary insights into GDM risk factors in this Greek population, and therefore do not constitute external validation of the present findings. Tranidou et al. [17] in a previous analysis of this cohort, demonstrated that maternal macronutrient intake patterns—particularly energy intake exceeding European Food Safety Authority (EFSA) recommendations (aOR = 1.99, 95% CI: 1.37–2.86) and increased protein and fiber intake during mid-gestation—were significantly associated with elevated GDM risk. In a separate analysis of the same population, Tranidou et al. [39] of the same population, examined pre-conception physical activity and confirmed that women who developed GDM were significantly older (mean age 34.4 vs. 32.0 years, *p* < 0.0001) and had higher pre-pregnancy BMI (median 24.6 vs. 22.7 kg/m^2^, *p* = 0.014). The identification of maternal age and BMI as dominant predictors in our current ML models is therefore consistent with earlier analyses of the same cohort and reflects the stable risk-factor structure of this population rather than independent replication. Additionally, Siargkas et al. [40], also using BORN2020 data, investigated macronutrient intake and LGA risk in women with GDM from the same BORN2020 cohort, further emphasizing the importance of nutritional factors in pregnancy outcomes among women with GDM.

### 4.3. Feature Importance and Clinical Relevance

The consistent identification of maternal age and pre-pregnancy BMI as top predictors aligns with established clinical knowledge and international guidelines [8,41]. An umbrella review of meta-analyses examining GDM risk factors confirmed that increased BMI and hypothyroidism show the strongest consistent evidence [42] for an association with GDM, supporting our feature importance findings. In our analysis, maternal age contributed 18.6% of predictive importance, while pre-pregnancy BMI contributed 6.7%. Collectively, the four demographic features accounted for 26.2% of model predictions. These findings support current screening recommendations that emphasize age ≥ 35 years and BMI ≥ 25 kg/m^2^ as key risk indicators warranting enhanced surveillance. Smoking was also more prevalent in the GDM group (17.9% vs. 8.8%), consistent with evidence that nicotine impairs insulin secretion and promotes insulin resistance, potentially contributing to altered glucose metabolism in early pregnancy. Logistic Regression, which achieved the best performance among tested algorithms, provides inherently interpretable coefficients. SHAP analysis further quantified individual feature contributions, yielding attributions that aligned well with clinical understanding, potentially facilitating acceptance in clinical practice.

The SHAP analysis provided insights into which features contributed most to the model’s discriminative predictions. However, SHAP values reflect predictive contribution within this specific model, not causal or independent biological effects, particularly given the use of unadjusted absolute intakes and the high collinearity among dietary features. The 22 dietary micronutrient features collectively accounted for 64.1% of total predictive importance, although this apparent dominance is largely attributable to the high feature count (22 micronutrients vs. 4 demographic features). On a per-feature basis, demographic predictors demonstrated more than double the individual importance of micronutrients (6.6% vs. 2.9% per feature), indicating that the micronutrient signal is distributed across many dietary variables rather than concentrated in a few strong predictors. This pattern is consistent with the high collinearity among dietary features, where individuals with higher intake of one micronutrient tend to have higher intake of others, reflecting overall diet quality and total energy intake.

Among individual micronutrients, calcium (9.3%), niacin (8.0%), and Vitamin B6 (7.7%) emerged as the most influential dietary predictors. These findings are partially consistent with our previous analysis of micronutrient intake in the same BORN2020 cohort [43], which identified significantly higher intakes of niacin (aOR = 1.07), iron (aOR = 1.19), manganese (aOR = 1.40), and zinc (aOR = 1.16) [43] in the GDM group compared to non-GDM controls. The concordance for niacin between traditional statistical associations and ML-derived SHAP feature importance strengthens the evidence for a role of this micronutrient in GDM pathophysiology.

The prominence of B-vitamins among the top micronutrient predictors (niacin at 8.0%, Vitamin B6 at 7.7%, folic acid at 7.5%, and thiamine at 5.8%, collectively accounting for approximately 29% of model importance) may reflect their interconnected roles in glucose metabolism and insulin sensitivity. Niacin is a precursor to NAD+/NADH, which are critical cofactors for glycolysis and oxidative phosphorylation, and pharmacological doses of niacin are known to induce insulin resistance [44]. Folic acid is essential for one-carbon metabolism, and its deficiency has been associated with insulin resistance [45,46]. The high ranking of calcium (9.3%, second highest among all features) is noteworthy, as calcium plays a role in insulin secretion from pancreatic beta cells and calcium homeostasis has been linked to glucose metabolism during pregnancy [47]. Emerging evidence suggests that adequate calcium intake may have a protective role against gestational diabetes, although findings remain heterogeneous across populations.

A critical interpretive caveat is that dietary micronutrients exhibit high collinearity—individuals with higher intake of one micronutrient tend to have higher intake of others—reflecting overall diet quality and total energy intake rather than independent nutrient effects. In Logistic Regression, multicollinearity can affect individual coefficient magnitudes while the overall model performance remains stable, which may explain the redistribution of importance across correlated micronutrient features. The 64.1% collective micronutrient importance likely reflects the distribution of a dietary pattern signal across 22 correlated features rather than 22 independent biological pathways. This is further supported by the observation that the five most influential micronutrients span different functional groups (mineral, B-vitamins, fat-soluble vitamin precursor), suggesting they may serve as proxies for broader dietary patterns rather than operating through independent mechanisms. The use of absolute (unadjusted) micronutrient intakes without energy adjustment is a key limitation. Without energy adjustment, the observed micronutrient SHAP importance likely reflects, at least in part, total energy intake acting as a confounder rather than independent nutrient-specific biological effects. This interpretation is further supported by the finding that the basic-only model (without any dietary features) achieved comparable or superior discriminative performance (Section 3.8), suggesting that the micronutrient signal may be largely redundant with information already captured by BMI and other demographic features that correlate with overall dietary intake. To address this, we conducted a sensitivity analysis using energy-adjusted micronutrient values computed via the Willett nutrient residual method across all 50 model configurations. Energy adjustment did not improve predictive performance across any model configuration; for the best-performing energy-adjusted configuration (MLP without resampling), the test AUC was 0.725 versus 0.720 for the same model with unadjusted inputs, confirming that the micronutrient signal captured by the models largely reflects overall dietary quantity rather than independent nutrient-specific effects. This is consistent with the finding that the basic nine-feature model without dietary features achieved comparable discriminative ability.

Although several micronutrients ranked highly in the SHAP importance analysis, this reflects a distributed predictive signal across a large number of correlated dietary variables rather than strong incremental predictive value. When model performance was evaluated directly, the addition of micronutrient features did not improve discrimination beyond the basic clinical model. Therefore, the cumulative SHAP importance of micronutrients is best interpreted as a proxy of overall dietary patterns or total energy intake rather than evidence of independent micronutrient-specific effects.

This finding is arguably one of the most clinically significant results of this study. The fact that a parsimonious nine-feature model using only routinely collected clinical data achieved comparable or superior discriminative performance to the full 31-feature model has two important implications. First, from a practical standpoint, early GDM risk stratification may not require detailed dietary assessment—a resource-intensive process involving validated FFQs, patient recall over six months, and nutrient calculation software—when standard demographic and clinical variables provide equivalent predictive value. Avoiding detailed FFQ collection in routine antenatal practice could save time and resources for both clinical staff and patients without impairing predictive performance. Second, from a methodological standpoint, the failure of micronutrient features to improve prediction suggests that their SHAP-derived importance in the full model reflects associations with total energy intake or overall dietary quality (which correlate with BMI and other demographic variables already in the model) rather than independent micronutrient-specific biological effects. This interpretation is further supported by the high collinearity among dietary features and the absence of energy adjustment, as discussed above.

### 4.4. Clinical Implications

It should be explicitly noted that this model is not suitable for direct clinical deployment without external validation and formal recalibration (e.g., Platt scaling or isotonic regression), as the calibration analysis revealed substantive miscalibration that precludes reliable absolute risk estimation.

From a clinical perspective, the discriminative performance of our models suggests potential utility as a screening tool to identify women who warrant enhanced monitoring.

At the pre-specified cross-validation-derived threshold, our best model achieved 78.3% sensitivity and 49.6% specificity, correctly identifying over three-quarters of GDM cases, although at the cost of reduced specificity. The high negative predictive value (0.932) observed for the best-performing model suggests potential utility for identifying women at very low GDM risk. However, this high NPV is partly a consequence of the moderate baseline prevalence (14.7%); even a model with modest sensitivity will produce high NPV when the majority of the population is disease-free. The clinical value therefore lies in the incremental improvement over applying no model (where NPV based on prevalence alone would be 85.3%), and in the model’s ability to identify a subgroup with meaningfully lower residual risk (6.8% missed GDM rate vs. 14.4% baseline). This characteristic is valuable in Mediterranean populations with moderate baseline GDM prevalence (14.7% in our cohort), where most women will not develop GDM and benefit most from reassurance and standard antenatal care pathways. Different probability thresholds may support different clinical use-cases rather than a single optimal decision point. A high-sensitivity threshold could be used in a rule-out pathway to identify women at very low risk who may safely continue routine antenatal care without intensified early monitoring. Conversely, a high-specificity threshold could define a smaller high-risk subgroup for targeted early lifestyle intervention, early biochemical testing, or closer metabolic surveillance. The selection of the operating threshold should therefore depend on the intended clinical application and available resources rather than a single global performance metric.

Decision curve analysis demonstrated that the model provides positive net benefit over the ‘treat all’ strategy within a narrow threshold range (approximately 12.5–14.5%) surrounding the observed prevalence. This narrow useful range reflects the model’s moderate discriminative ability and indicates that the clinical utility is primarily confined to the decision context where the threshold of action coincides with the population GDM prevalence. At higher thresholds, the model continues to offer marginal advantage over universal screening but with limited absolute net benefit. These findings reinforce the need for recalibration and external validation before the model can be recommended for clinical risk stratification.

Furthermore, the modest performance of our model compared to studies utilizing biochemical markers (e.g., fasting glucose, HbA1c) highlights a fundamental distinction in screening [24,25,26]. Physiological markers offer a ‘proximal’ and direct readout of metabolic status, whereas dietary intake is a ‘distal’ and noisy signal. Self-reported intake does not equate to physiological bioavailability, and as our results demonstrate, may not capture the metabolic dysregulation necessary for GDM development as accurately as direct serum markers.

### 4.5. Strengths and Limitations

This study has several notable strengths. The BORN2020 cohort provides prospectively collected, high-quality data from a well-characterized Mediterranean population with standardized GDM diagnosis using HSOG criteria based on IADPSG/HAPO thresholds [31]. The comprehensive dietary assessment using validated food frequency questionnaires allowed examination of nutritional features rarely available in prediction model development. Our systematic comparison of ten ML algorithms across five sampling strategies provides robust evidence regarding optimal modeling strategies for this clinical question. Additionally, the strict restriction to features collected at the first-trimester visit ensures temporal validity and clinical applicability—a methodological consideration often overlooked in prediction model studies that inadvertently include post-outcome information. As far as we are aware, this represents the most extensive investigation regarding machine learning-based GDM prediction conducted in a Greek population. The generalization analysis constitutes a major strength of this study. The small or negative cross-validation to test set gaps observed for the best-performing models (e.g., −0.018 for Logistic Regression) indicate low optimism and increase confidence that the reported performance estimates reflect real-world clinical applicability rather than inflated training performance.

The calibration analysis revealed substantive miscalibration: the intercept of −1.83 indicates systematic underestimation of GDM risk, while the slope of 0.56 indicates that predicted probabilities are overly dispersed. Although poor calibration is common in prediction models developed on limited datasets, these results indicate that the model’s predicted probabilities should not be used directly for clinical decision-making without formal recalibration (e.g., Platt scaling or isotonic regression) in an independent cohort. Importantly, poor calibration does not invalidate the model’s discriminative ability (AUC), which reflects the model’s capacity to rank patients by risk; however, it underscores the distinction between discrimination and calibration, and the need for both to be adequate before clinical implementation.

With 117 GDM events and 31 predictors, the events-per-variable ratio (EPV) was approximately 3.8—well below the conventional threshold of 10—which constrains model complexity and favors regularized linear approaches, consistent with the observed outperformance of Logistic Regression over gradient boosting methods. While recent evidence suggests that rigid EPV thresholds may be overly conservative for prediction-focused models with regularization [33,48], the low EPV increases the risk of model instability and overfitting, particularly for coefficient estimation and individual feature importance values. L2 (ridge) regularization was applied to the Logistic Regression model, which stabilizes coefficient estimates and mitigates the effects of low EPV, and the comparable performance of the nine-feature basic model supports the view that the 31-feature model did not substantially overfit to training data. Nevertheless, the low EPV underscores the exploratory nature of this study and the critical need for external validation before any clinical implementation. Future studies should also consider dimensionality reduction approaches (e.g., principal component analysis or feature clustering) to improve the EPV ratio while preserving clinically meaningful feature groups.

## 5. Conclusions

In conclusion, this exploratory, development-phase study demonstrates that ML models utilizing first-trimester clinical data achieve fair discriminative ability (best AUC ≈ 0.66) for early GDM prediction in a Greek Mediterranean cohort, with high negative predictive value (>0.93) driven largely by baseline prevalence. Detailed FFQ-derived micronutrient data did not improve predictive performance beyond basic clinical variables, supporting a parsimonious approach to early-pregnancy risk stratification. However, substantial miscalibration, a low events-per-variable ratio, and the absence of external validation limit the clinical applicability of these models in their current form. Future work should prioritize external validation, probability recalibration, and evaluation of models incorporating early-pregnancy biochemical markers.

Taken together, these findings position the present work as an exploratory benchmarking study that informs feature selection, modeling strategy, and validation requirements for future externally validated early-pregnancy GDM prediction models.

## Figures and Tables

**Table 1:** Baseline Characteristics of Study Population.

Variable	Non-GDM (*n =* 680)	GDM (*n* = 117)	*p*-Value
**Demographics**			
Maternal age (years), mean ± SD	32.1 ± 4.9	34.2 ± 4.5	<0.001
Advanced maternal age (≥35 years), n (%)	186 (27.4)	51 (43.6)	<0.001
**Anthropometric Measurements**			
Pre-pregnancy BMI (kg/m^2^), median [IQR]	22.7 [20.8–26.0]	23.7 [21.7–28.5]	0.004
Normal weight, n (%)	438 (64.4)	69 (59.0)	-
Overweight, n (%)	139 (20.4)	21 (17.9)	-
Obese, n (%)	75 (11.0)	25 (21.4)	-
Pre-pregnancy weight (kg), mean ± SD	66.1 ± 13.8	70.8 ± 17.4	0.001
Height (cm), mean ± SD	165.9 ± 6.0	165.4 ± 6.4	0.416
**Clinical Characteristics**			
Parity, n (%)			0.971
0	347 (51.0)	60 (51.3)	-
1	253 (37.2)	44 (37.6)	-
2	69 (10.1)	12 (10.3)	-
3	9 (1.3)	1 (0.9)	-
4	2 (0.3)	0 (0.0)	-
Assisted reproductive technology, n (%)	48 (7.1)	11 (9.4)	0.482
Thyroid disease, n (%)	93 (13.7)	13 (11.1)	0.544
**Lifestyle Factors**			
Walking > 10 min/day (times/week), median [IQR]	0 [0, 1]	0 [0, 1]	0.433
Current smoking, n (%)	60 (8.8)	21 (17.9)	0.004

Continuous variables are presented as mean ± standard deviation (SD) for normally distributed data or median [interquartile range (IQR)] for non-normally distributed data. Categorical variables are presented as a number (percentage). Normality was assessed using the Shapiro–Wilk test. Between-group comparisons were performed using the independent *t*-test for normally distributed continuous variables, the Mann–Whitney *U* test for non-normally distributed continuous variables, and the chi-square test or Fisher’s exact test for categorical variables, as appropriate. *p*-values are reported for overall group comparisons; category-specific *p*-values were not calculated, as only overall group comparisons were performed for multi-level categorical variables; sub-category rows are indicated with "–". Advanced maternal age was defined as ≥35 years. Body mass index (BMI) categories were defined according to World Health Organization criteria (normal weight: 18.5–24.9 kg/m^2^; overweight: 25.0–29.9 kg/m^2^; obese: ≥30.0 kg/m^2^). Walking frequency reflects self-reported walking activity of more than 10 min per day. Statistical significance was defined as *p* < 0.05. For categorical variables with multiple levels (e.g., Parity), the *p*-value represents the overall chi-square test for independence.

**Table 2:** Top 10 Model Configurations Ranked by AUC-ROC on Independent Test Set.

Rank	Model	Sampler	AUC (95% CI)	Sensitivity (95% CI)	Specificity (95% CI)	PPV (95% CI)	NPV (95% CI)	F1	Brier
1	Logistic Regression	None	0.664 (0.542–0.777)	0.783 (0.588–0.941)	0.496 (0.413–0.582)	0.207 (0.122–0.307)	0.932 (0.867–0.986)	0.327	0.250
2	MLP	None	0.662 (0.538–0.776)	0.783 (0.583–0.950)	0.533 (0.450–0.617)	0.220 (0.130–0.316)	0.936 (0.877–0.986)	0.343	0.168
3	Logistic Regression	ADASYN	0.661 (0.536–0.774)	0.696 (0.500–0.875)	0.657 (0.579–0.735)	0.254 (0.143–0.367)	0.928 (0.874–0.971)	0.372	0.250
4	LightGBM	None	0.650 (0.513–0.779)	0.391 (0.192–0.591)	0.912 (0.858–0.957)	0.429 (0.217–0.643)	0.899 (0.847–0.949)	0.409	0.115
5	LightGBM	ADASYN	0.639 (0.515–0.757)	0.348 (0.150–0.565)	0.912 (0.862–0.957)	0.400 (0.188–0.619)	0.893 (0.838–0.943)	0.372	0.152
6	Extra Trees	None	0.638 (0.507–0.760)	0.522 (0.318–0.722)	0.788 (0.716–0.851)	0.293 (0.156–0.438)	0.908 (0.855–0.958)	0.375	0.118
7	Logistic Regression	SMOTE-Tomek	0.636 (0.506–0.757)	0.696 (0.500–0.875)	0.613 (0.527–0.693)	0.232 (0.132–0.333)	0.923 (0.866–0.970)	0.348	0.262
8	LightGBM	Borderline SMOTE	0.628 (0.494–0.753)	0.391 (0.200–0.591)	0.883 (0.826–0.934)	0.360 (0.182–0.556)	0.896 (0.844–0.947)	0.375	0.130
9	AdaBoost	None	0.628 (0.477–0.775)	0.435 (0.227–0.643)	0.869 (0.813–0.925)	0.357 (0.182–0.539)	0.902 (0.853–0.953)	0.392	0.187
10	XGBoost	ADASYN	0.627 (0.499–0.752)	0.478 (0.261–0.682)	0.796 (0.730–0.859)	0.282 (0.146–0.422)	0.901 (0.848–0.952)	0.355	0.148

Sampler ‘None’ indicates no resampling was applied. All metrics evaluated on the independent 20% test set (*n* = 160). Classification threshold pre-specified from cross-validation (average Youden’s index across 5 CV folds = 0.449) and applied to the independent internal hold-out test set. AUC-ROC is threshold-independent and unaffected. 95% CIs for all metrics were calculated using bootstrap resampling with 1000 iterations.

**Table 3:** Comparative Performance of Sampling Strategies Across All Algorithms.

Sampling Strategy	n	Mean AUC	SD	Best AUC	Best Model
None	10	0.619	±0.038	0.664	Logistic Regression
ADASYN	10	0.618	±0.021	0.661	Logistic Regression
SMOTE	10	0.608	±0.018	0.627	MLP
Borderline SMOTE	10	0.601	±0.021	0.628	LightGBM
SMOTE-Tomek	10	0.594	±0.024	0.636	Logistic Regression

Mean AUC ± SD represents the average performance across all 10 machine learning algorithms tested with each sampling strategy. n: Number of algorithm–sampler combinations tested. Best AUC indicates the highest performance achieved with that sampling strategy. All sampling strategies used a target minority class ratio of 0.6 and were applied only to the training set.

**Table 4:** Cross-Validation vs. Test Performance (Top 10 Models).

Model	Sampler	CV AUC	Test AUC	Gap	Overfitting
Logistic Regression	None	0.646	0.664	−0.018	Minimal
MLP	None	0.651	0.662	−0.011	Minimal
Logistic Regression	ADASYN	0.722	0.661	0.061	Moderate
LightGBM	None	0.625	0.650	−0.025	Minimal
LightGBM	ADASYN	0.936	0.639	0.297	Severe
Extra Trees	None	0.619	0.638	−0.019	Minimal
Logistic Regression	SMOTE-Tomek	0.704	0.636	0.068	Moderate
LightGBM	Borderline SMOTE	0.941	0.628	0.313	Severe
AdaBoost	None	0.631	0.628	0.003	Minimal
XGBoost	ADASYN	0.938	0.627	0.311	Severe

Gap = CV AUC − Test AUC. Overfitting categories: Minimal (<5% gap), Moderate (5–15% gap), Severe (>15% gap). Overfitting was categorized as minimal (<5% gap), moderate (5–15% gap), or severe (>15% gap) based on established conventions for assessing model optimism in clinical prediction studies [37]. CV AUC: mean AUC from 5-fold stratified cross-validation on the training set.

**Table 5:** Best Performance Per Algorithm.

Algorithm	Sampler	AUC-ROC	95% CI	Sens	Spec	F1
Logistic Regression	None	0.664	(0.542–0.777)	0.739	0.620	0.370
MLP	None	0.662	(0.538–0.776)	0.783	0.533	0.343
LightGBM	None	0.650	(0.513–0.779)	0.391	0.912	0.409
Extra Trees	None	0.638	(0.507–0.760)	0.522	0.788	0.375
AdaBoost	None	0.628	(0.477–0.775)	0.435	0.869	0.392
XGBoost	ADASYN	0.627	(0.499–0.752)	0.478	0.796	0.355
Gradient Boosting	Borderline SMOTE	0.626	(0.499–0.741)	0.565	0.708	0.342
Random Forest	ADASYN	0.619	(0.482–0.745)	0.696	0.577	0.330
Bagging	SMOTE-Tomek	0.615	(0.494–0.723)	0.565	0.701	0.338
CatBoost	None	0.608	(0.458–0.743)	0.391	0.891	0.383

Results show the best-performing hyperparameter configuration for each algorithm family. AUC-ROC: Area under the receiver operating characteristic curve. CI: Confidence interval (95%, calculated using bootstrap resampling with 1000 iterations). Hyperparameters were optimized using exhaustive grid search with 5-fold stratified cross-validation on the training set (*n* = 637, 80% of data). Final performance metrics were evaluated on an independent test set (*n* = 160, 20% of data). Sampling strategy indicates the class imbalance handling method applied during training (None = no resampling; SMOTE = Synthetic Minority Over-sampling Technique; SMOTE-Tomek = combined over- and under-sampling).

**Table 6:** Feature Importance Ranking Based on SHAP Analysis of the Logistic Regression Model (Top 15).

Rank	Feature	Category	Mean |SHAP|	Contribution (%)
1	Maternal Age	Demographic	0.090	18.6
2	Calcium	Micronutrient	0.045	9.3
3	Niacin	Micronutrient	0.039	8.0
4	Vitamin B6	Micronutrient	0.037	7.7
5	Folic Acid (DFE)	Micronutrient	0.036	7.5
6	Pre-pregnancy BMI	Demographic	0.032	6.7
7	Retinol	Micronutrient	0.029	6.1
8	Thiamine (B1)	Micronutrient	0.028	5.8
9	Magnesium	Micronutrient	0.018	3.7
10	Parity	Obstetric	0.017	3.4
11	Vitamin C	Micronutrient	0.015	3.1
12	Smoking Status	Clinical	0.014	2.8
13	Manganese	Micronutrient	0.013	2.8
14	Assisted Reproduction	Clinical	0.011	2.2
15	Potassium	Micronutrient	0.010	2.0

SHAP: SHapley Additive exPlanations. Mean |SHAP| represents the average absolute SHAP value across all test set predictions (*n* = 160). Contribution (%) = feature’s mean |SHAP|/sum of all mean |SHAP| values × 100. SHAP values were computed using KernelExplainer with k-means background summarization (k = 50). Verification using LinearExplainer (exact for linear models) produced a highly consistent feature ranking (Spearman ρ = 0.982, *p* < 0.001; Supplementary Table S4).

**Table 7:** Category-Level Feature Importance Summary.

Category	N Features	Total Importance (%)	Per-Feature Average (%)	Top Feature
Demographic	4	26.2	6.6	Maternal Age
Micronutrient	22	64.1	2.9	Calcium
Clinical	3	6.1	2.0	Smoking Status
Obstetric	1	3.4	3.4	Parity
Lifestyle	1	0.1	0.1	Walking Frequency

Total Importance (%) = sum of individual feature contributions within each category. Per-Feature Average (%) = Total Importance/N Features, representing the average contribution per individual feature. This metric accounts for the disparity in feature counts across categories (22 micronutrients vs. 4 demographic features).

**Table 8:** Threshold analysis table.

Method	Threshold	Sensitivity (95% CI)	Specificity (95% CI)	PPV (95% CI)	NPV (95% CI)
CV-derived Youden’s Index	0.449	0.783 (0.588–0.941)	0.496 (0.413–0.582)	0.207 (0.122–0.307)	0.932 (0.867–0.986)
Sensitivity ≈ 80%	0.464	0.783 (0.588–0.941)	0.540 (0.457–0.628)	0.222 (0.132–0.325)	0.937 (0.878–0.987)
Sensitivity ≈ 90%	0.365	0.913 (0.782–1.000)	0.307 (0.231–0.387)	0.181 (0.110–0.254)	0.955 (0.879–1.000)

## Data Availability

The data presented in this study are available on reasonable request from the corresponding author. The data are not publicly available due to privacy restrictions.

## Supplementary Materials

The following supporting information can be downloaded at: https://www.mdpi.com/article/10.3390/jcm15062461/s1, Table S1: Complete Results of All 50 Model Configurations (10 Algorithms × 5 Sampling Strategies), Ranked by Test Set AUC-ROC; Table S2: Hyperparameter Search Spaces Explored for Each Algorithm; Table S3: Optimal Hyperparameters Identified for the Best-Performing Configuration of Each Algorithm; Table S4: Complete SHAP Feature Importance Ranking for All 31 Features (Logistic Regression Model, AUC = 0.664); Table S5: Feature Definitions, Units, Variable Types, and Clinical Relevance to GDM; Table S6: Comparison of SHAP feature importance rankings between LinearExplainer and KernelExplainer for the Logistic Regression model; Text S1: Feature Selection Rationale; Figure S1: Comparison of SHAP feature importance rankings derived from LinearExplainer and KernelExplainer for the best-performing Logistic Regression model.
