# Supplementary material for: First-Trimester Gestational Diabetes Mellitus Risk Prediction with Machine Learning Techniques: Results from the BORN2020 Cohort Study
Source: J Clin Med. 2026 Mar 23;15(6):2461. doi: 10.3390/jcm15062461 (PMC13027101; doi:10.3390/jcm15062461)

Supplementary Materials

Machine Learning-Based Early Prediction of Gestational Diabetes Mellitus Risk Using Data from First Trimester: Results from the BORN2020 Cohort Study

Table S1. Complete Results of All 50 Model Configurations (10 Algorithms × 5 Sampling Strategies), Ranked by Test Set AUC-ROC.

Rank	Algorithm	Sampling	AUC	95% CI	CV AUC (±SD)	Sens	Spec	Prec	NPV	F1	Acc	Brier
1	LogisticRegression	No resampling	0.664	0.542 – 0.777	0.646 ± 0.036	0.739	0.620	0.246	0.934	0.370	0.637	0.2499
2	MLP	No resampling	0.662	0.538 – 0.776	0.651 ± 0.036	0.783	0.533	0.220	0.936	0.343	0.569	0.1683
3	LogisticRegression	ADASYN	0.661	0.536 – 0.774	0.722 ± 0.038	0.696	0.657	0.254	0.928	0.372	0.662	0.2499
4	LightGBM	No resampling	0.650	0.513 – 0.779	0.625 ± 0.050	0.391	0.912	0.429	0.899	0.409	0.838	0.1153
5	LightGBM	ADASYN	0.638	0.515 – 0.757	0.936 ± 0.012	0.348	0.912	0.400	0.893	0.372	0.831	0.1520
6	ExtraTrees	No resampling	0.638	0.507 – 0.760	0.619 ± 0.058	0.522	0.788	0.293	0.908	0.375	0.750	0.1182
7	LogisticRegression	SMOTE-Tomek	0.636	0.506 – 0.757	0.704 ± 0.039	0.696	0.613	0.232	0.923	0.348	0.625	0.2616
8	LightGBM	BorderlineSMOTE	0.628	0.494 – 0.753	0.941 ± 0.011	0.391	0.883	0.360	0.896	0.375	0.812	0.1297
9	AdaBoost	No resampling	0.628	0.477 – 0.775	0.631 ± 0.045	0.435	0.869	0.357	0.902	0.392	0.806	0.1869
10	XGBoost	ADASYN	0.627	0.499 – 0.752	0.938 ± 0.012	0.478	0.796	0.282	0.901	0.355	0.750	0.1480
11	MLP	SMOTE	0.627	0.499 – 0.747	0.856 ± 0.061	0.391	0.861	0.321	0.894	0.353	0.794	0.1825
12	LogisticRegression	SMOTE	0.627	0.490 – 0.754	0.703 ± 0.058	0.609	0.679	0.241	0.912	0.346	0.669	0.2623
13	GradientBoosting	BorderlineSMOTE	0.626	0.499 – 0.741	0.948 ± 0.008	0.565	0.708	0.245	0.907	0.342	0.688	0.1278
14	LightGBM	SMOTE	0.625	0.498 – 0.743	0.945 ± 0.019	0.826	0.453	0.202	0.939	0.325	0.506	0.1489

Rank	Algorithm	Sampling	AUC	95% CI	CV AUC (±SD)	Sens	Spec	Prec	NPV	F1	Acc	Brier
15	ExtraTrees	ADASYN	0.625	0.495 – 0.742	0.958 ± 0.014	0.739	0.562	0.221	0.928	0.340	0.588	0.1351
16	RandomForest	ADASYN	0.619	0.482 – 0.745	0.942 ± 0.009	0.696	0.577	0.216	0.919	0.330	0.594	0.1364
17	ExtraTrees	SMOTE	0.616	0.487 – 0.733	0.965 ± 0.015	0.739	0.511	0.202	0.921	0.318	0.544	0.1291
18	LogisticRegression	BorderlineSMOTE	0.615	0.475 – 0.745	0.733 ± 0.015	0.609	0.635	0.219	0.906	0.322	0.631	0.2631
19	Bagging	SMOTE-Tomek	0.615	0.494 – 0.723	0.948 ± 0.036	0.565	0.701	0.241	0.906	0.338	0.681	0.1355
20	XGBoost	No resampling	0.614	0.480 – 0.742	0.615 ± 0.055	0.391	0.905	0.409	0.899	0.400	0.831	0.1225
21	ExtraTrees	BorderlineSMOTE	0.612	0.484 – 0.723	0.958 ± 0.013	0.652	0.620	0.224	0.914	0.333	0.625	0.1286
22	Bagging	ADASYN	0.612	0.484 – 0.729	0.944 ± 0.011	0.391	0.847	0.300	0.892	0.340	0.781	0.1355
23	XGBoost	BorderlineSMOTE	0.609	0.475 – 0.731	0.939 ± 0.015	0.609	0.672	0.237	0.911	0.341	0.662	0.1493
24	CatBoost	No resampling	0.608	0.458 – 0.743	0.632 ± 0.051	0.391	0.891	0.375	0.897	0.383	0.819	0.1181
25	XGBoost	SMOTE	0.607	0.480 – 0.728	0.946 ± 0.022	0.522	0.723	0.240	0.900	0.329	0.694	0.1565
26	Bagging	SMOTE	0.607	0.475 – 0.724	0.952 ± 0.020	0.565	0.693	0.236	0.905	0.333	0.675	0.1354
27	AdaBoost	BorderlineSMOTE	0.605	0.462 – 0.739	0.878 ± 0.019	0.522	0.737	0.250	0.902	0.338	0.706	0.2249
28	MLP	SMOTE-Tomek	0.604	0.454 – 0.734	0.867 ± 0.047	0.522	0.796	0.300	0.908	0.381	0.756	0.1711
29	CatBoost	ADASYN	0.603	0.475 – 0.733	0.943 ± 0.012	0.870	0.372	0.189	0.944	0.310	0.444	0.1374
30	MLP	ADASYN	0.603	0.451 – 0.736	0.897 ± 0.050	0.435	0.869	0.357	0.902	0.392	0.806	0.1640
31	RandomForest	SMOTE-Tomek	0.603	0.471 – 0.720	0.950 ± 0.031	0.565	0.679	0.228	0.903	0.325	0.662	0.1355

Rank	Algorithm	Sampling	AUC	95% CI	CV AUC (±SD)	Sens	Spec	Prec	NPV	F1	Acc	Brier
32	AdaBoost	SMOTE	0.602	0.443 – 0.750	0.879 ± 0.023	0.391	0.934	0.500	0.901	0.439	0.856	0.2230
33	RandomForest	SMOTE	0.601	0.471 – 0.720	0.948 ± 0.021	0.391	0.810	0.257	0.888	0.310	0.750	0.1352
34	GradientBoosting	SMOTE-Tomek	0.600	0.485 – 0.702	0.955 ± 0.025	0.696	0.533	0.200	0.912	0.311	0.556	0.1502
35	RandomForest	No resampling	0.599	0.462 – 0.724	0.605 ± 0.065	0.565	0.650	0.213	0.899	0.310	0.637	0.1204
36	GradientBoosting	SMOTE	0.597	0.471 – 0.712	0.950 ± 0.016	0.870	0.343	0.182	0.940	0.301	0.419	0.1417
37	AdaBoost	ADASYN	0.596	0.452 – 0.739	0.861 ± 0.014	0.391	0.854	0.310	0.893	0.346	0.787	0.2260
38	AdaBoost	SMOTE-Tomek	0.595	0.426 – 0.748	0.868 ± 0.028	0.391	0.942	0.529	0.902	0.450	0.863	0.2232
39	GradientBoosting	ADASYN	0.595	0.454 – 0.726	0.946 ± 0.009	0.435	0.825	0.294	0.897	0.351	0.769	0.1330
40	Bagging	No resampling	0.592	0.452 – 0.717	0.621 ± 0.074	0.391	0.825	0.273	0.890	0.321	0.762	0.1242
41	MLP	BorderlineSMOTE	0.588	0.437 – 0.718	0.915 ± 0.020	0.522	0.737	0.250	0.902	0.338	0.706	0.1695
42	CatBoost	BorderlineSMOTE	0.587	0.451 – 0.710	0.942 ± 0.011	0.870	0.328	0.179	0.938	0.296	0.406	0.1342
43	ExtraTrees	SMOTE-Tomek	0.583	0.456 – 0.697	0.967 ± 0.028	0.696	0.518	0.195	0.910	0.305	0.544	0.1339
44	RandomForest	BorderlineSMOTE	0.583	0.455 – 0.700	0.943 ± 0.011	0.391	0.796	0.243	0.886	0.300	0.738	0.1384
45	XGBoost	SMOTE-Tomek	0.580	0.449 – 0.703	0.949 ± 0.022	0.652	0.547	0.195	0.904	0.300	0.562	0.1523
46	CatBoost	SMOTE	0.570	0.438 – 0.692	0.947 ± 0.016	0.261	0.905	0.316	0.879	0.286	0.812	0.1372
47	LightGBM	SMOTE-Tomek	0.569	0.437 – 0.696	0.947 ± 0.028	0.348	0.825	0.250	0.883	0.291	0.756	0.1539
48	Bagging	BorderlineSMOTE	0.561	0.424 – 0.681	0.942 ± 0.011	0.348	0.818	0.242	0.882	0.286	0.750	0.1429

Rank	Algorithm	Sampling	AUC	95% CI	CV AUC (±SD)	Sens	Spec	Prec	NPV	F1	Acc	Brier
49	CatBoost	SMOTE-Tomek	0.554	0.414 – 0.688	0.955 ± 0.023	0.565	0.642	0.210	0.898	0.306	0.631	0.1360
50	GradientBoosting	No resampling	0.539	0.383 – 0.687	0.642 ± 0.068	0.565	0.657	0.217	0.900	0.313	0.644	0.1212

AUC: Area under the receiver operating characteristic curve. 95% CI: Bootstrap confidence interval (1,000 iterations). CV AUC: Mean AUC from 5-fold stratified cross-validation on the training set (± standard deviation). Sens: Sensitivity; Spec: Specificity; Prec: Positive Predictive Value; NPV: Negative Predictive Value; F1: F1-Score; Acc: Accuracy; Brier: Brier Score. All metrics evaluated on the independent 20% hold-out test set (n = 160; 23 GDM, 137 non-GDM). Classification threshold optimized using Youden's Index. Sampling strategies applied a target minority class ratio of 0.6 to the training set only. Note: The above 50 configurations used the full 31-feature set. A separately evaluated reduced model using only 9 demographic and clinical features (maternal age, pre-pregnancy weight, height, BMI, parity, ART, thyroid disease, smoking, walking activity) achieved a numerically higher test set AUC of 0.712 (95% CI: 0.589–0.825) using MLP without resampling. Full results of this analysis are reported in Section 3.8 of the main text.

Table S2. Hyperparameter Search Spaces Explored for Each Algorithm.

Algorithm	Hyperparameter	Search Space	N
Logistic Regression	C	[0.001, 0.01, 0.1, 1, 10, 100]	6
Logistic Regression	penalty	['l1', 'l2']	2
Logistic Regression	solver	['liblinear', 'saga']	2
Logistic Regression	class_weight	[None, 'balanced']	2
Logistic Regression	max_iter	[5000]	1
Random Forest	n_estimators	[100, 200, 300]	3
Random Forest	max_depth	[3, 5, 10, None]	4
Random Forest	min_samples_split	[2, 5, 10]	3
Random Forest	min_samples_leaf	[1, 2, 4]	3
Random Forest	class_weight	[None, 'balanced']	2
Extra Trees	n_estimators	[200, 300]	2
Extra Trees	max_depth	[None]	1
Extra Trees	min_samples_split	[2, 5]	2
Extra Trees	min_samples_leaf	[1]	1
Extra Trees	class_weight	[None, 'balanced']	2
XGBoost	n_estimators	[100, 200]	2
XGBoost	max_depth	[5, 7]	2
XGBoost	learning_rate	[0.05, 0.1]	2
XGBoost	subsample	[0.7, 0.8]	2
XGBoost	colsample_bytree	[0.7, 0.8]	2
XGBoost	scale_pos_weight	[1, 5]	2
LightGBM	n_estimators	[100, 200, 500]	3
LightGBM	max_depth	[-1]	1
LightGBM	learning_rate	[0.05, 0.1]	2
LightGBM	num_leaves	[31]	1
LightGBM	colsample_bytree	[0.8]	1
LightGBM	subsample	[0.8]	1
LightGBM	reg_alpha	[0, 0.1]	2
LightGBM	reg_lambda	[0]	1
CatBoost	iterations	[300]	1
CatBoost	depth	[5, 7]	2
CatBoost	learning_rate	[0.05]	1
CatBoost	l2_leaf_reg	[3, 5]	2
Gradient Boosting	n_estimators	[100, 200]	2
Gradient Boosting	max_depth	[7]	1
Gradient Boosting	learning_rate	[0.01, 0.1]	2
Gradient Boosting	subsample	[0.7, 0.8]	2
AdaBoost	n_estimators	[50, 200]	2
AdaBoost	learning_rate	[1.0]	1

Algorithm	Hyperparameter	Search Space	N
Bagging	n_estimators	[50, 100, 200]	3
Bagging	max_samples	[0.7, 1.0]	2
Bagging	max_features	[0.5]	1
MLP	hidden_layer_sizes	[(200,), (100,50,25), (200,100,50)]	8
MLP	alpha	[0.0001, 0.001, 0.01]	3
MLP	activation	['relu', 'tanh']	2
MLP	learning_rate	['adaptive']	1
MLP	early_stopping	[True]	1
MLP	max_iter	[2000]	1

Hyperparameters were optimized using exhaustive grid search with 5-fold stratified cross-validation on the training set (n = 637). CatBoost was tuned using manual cross-validation due to incompatibility with scikit-learn's GridSearchCV in version 1.6+. MLP: Multi-Layer Perceptron. N: Number of distinct values explored.

Table S3. Optimal Hyperparameters Identified for the Best-Performing Configuration of Each Algorithm.

Algorithm	Best Sampling	AUC	Optimal Hyperparameters
Logistic Regression	No resampling	0.664	C=0.1, class_weight='balanced', penalty='l2', solver='saga'
MLP	No resampling	0.662	activation='tanh', alpha=0.0001, hidden_layer_sizes=(200,), learning_rate='adaptive', early_stopping=True
LightGBM	No resampling	0.650	colsample_bytree=0.8, learning_rate=0.05, max_depth=-1, n_estimators=100, num_leaves=31, reg_alpha=0.1, subsample=0.8
Extra Trees	No resampling	0.638	class_weight='balanced', max_depth=None, min_samples_leaf=1, min_samples_split=2, n_estimators=200
AdaBoost	No resampling	0.628	learning_rate=1.0, n_estimators=50
XGBoost	ADASYN	0.627	colsample_bytree=0.7, learning_rate=0.05, max_depth=7, n_estimators=200, scale_pos_weight=5, subsample=0.8
Gradient Boosting	Bord.SMOTE	0.626	learning_rate=0.1, max_depth=7, n_estimators=200, subsample=0.8
Random Forest	ADASYN	0.619	class_weight='balanced', max_depth=None, min_samples_leaf=1, min_samples_split=2, n_estimators=300
CatBoost	No resampling	0.608	iterations=300, depth=5, learning_rate=0.05, l2_leaf_reg=3
Bagging	SMOTE-Tomek	0.615	max_features=0.5, max_samples=1.0, n_estimators=200

Hyperparameters were selected based on maximum cross-validation AUC-ROC from 5-fold stratified cross-validation on the training set (n = 637). Algorithms are ranked by their best test set AUC-ROC. Bord.SMOTE: Borderline SMOTE.

Table S4. Complete SHAP Feature Importance Ranking for All 31 Features (Logistic Regression Model, AUC = 0.664).

Rank	Feature	Category	Mean |SHAP|	Contrib. (%)	Cumul. (%)
1	Maternal Age	Demographic	0.0896	18.6	18.6
2	Calcium	Micronutrient	0.0449	9.3	27.9
3	Niacin	Micronutrient	0.0387	8.0	35.9
4	Vitamin B6	Micronutrient	0.0371	7.7	43.6
5	Folic Acid (DFE)	Micronutrient	0.0359	7.5	51.1
6	Pre-pregnancy BMI	Demographic	0.0322	6.7	57.8
7	Retinol	Micronutrient	0.0295	6.1	63.9
8	Thiamine (B1)	Micronutrient	0.0276	5.8	69.7
9	Magnesium	Micronutrient	0.0177	3.7	73.4
10	Parity	Obstetric	0.0165	3.4	76.8
11	Vitamin C	Micronutrient	0.0147	3.1	79.9
12	Smoking Status	Clinical	0.0136	2.8	82.7
13	Manganese	Micronutrient	0.0132	2.8	85.5
14	Assisted Reproduction	Clinical	0.0108	2.2	87.7
15	Potassium	Micronutrient	0.0096	2.0	89.7
16	Vitamin K	Micronutrient	0.0080	1.7	91.4
17	Vitamin E	Micronutrient	0.0054	1.1	92.5
18	Zinc	Micronutrient	0.0053	1.1	93.6
19	Thyroid Condition	Clinical	0.0051	1.1	94.7
20	Vitamin D	Micronutrient	0.0048	1.0	95.7
21	Copper	Micronutrient	0.0036	0.8	96.5
22	Pre-pregnancy Weight	Demographic	0.0035	0.7	97.2
23	Riboflavin (B2)	Micronutrient	0.0033	0.7	97.9
24	Iodine	Micronutrient	0.0032	0.7	98.6
25	Vitamin A	Micronutrient	0.0029	0.6	99.2
26	Phosphorus	Micronutrient	0.0010	0.2	99.4
27	Height	Demographic	0.0010	0.2	99.6
28	Sodium	Micronutrient	0.0007	0.1	99.7
29	Iron	Micronutrient	0.0006	0.1	99.8
30	Walking Frequency	Lifestyle	0.0003	0.1	99.9
31	Vitamin B12	Micronutrient	0.0002	0.0	99.9

SHAP: SHapley Additive exPlanations. Mean |SHAP| represents the average absolute SHAP value across all test set predictions (n = 160). Contribution (%) = feature's mean |SHAP| / sum of all mean |SHAP| values × 100. Cumul.: Cumulative contribution percentage. SHAP values were computed using KernelExplainer with k-means summarized background data (k = 50) on the best-performing Logistic Regression model with no resampling. Dietary micronutrient features represent absolute pre-pregnancy intakes assessed via a validated FFQ. Given the low events-per-variable ratio (~3.8), SHAP feature rankings should be interpreted as exploratory and hypothesis-generating rather than stable estimates of predictor importance; rankings may be sensitive to sampling variability in this low-event setting. See main text Section 4.5 for full discussion.

Table S5. Feature Definitions, Units, Variable Types, and Clinical Relevance to GDM.

Display Name	Column Name	Category	Unit	Type	Clinical Relevance to GDM
Maternal Age	MA	Demographic	years	Continuous	Advanced maternal age (≥35) is a well-established GDM risk factor
Pre-pregnancy Weight	Wt	Demographic	kg	Continuous	Higher pre-pregnancy weight associated with increased GDM risk
Height	Ht	Demographic	cm	Continuous	Included for BMI interpretation context
Pre-pregnancy BMI	BMIpre	Demographic	kg/m^2	Continuous	Pre-pregnancy obesity (BMI ≥30) is among strongest GDM risk factors
Assisted Reproduction	ART	Clinical	0/1	Binary	Assisted reproduction associated with metabolic alterations
Thyroid Condition	Thyroid	Clinical	0/1	Binary	Thyroid dysfunction linked to glucose metabolism impairment
Smoking Status	Smoking	Clinical	0/1	Binary	Conflicting evidence; may modify insulin resistance
Parity	Parity	Obstetric	count	Discrete	Multiparity associated with GDM recurrence
Walking Frequency	Walking - A	Lifestyle	times/wk	Continuous	Physical activity is a modifiable protective factor for GDM
Vitamin A	Vit. A - A	Micronutrient	μg	Continuous	Antioxidant; retinol metabolism linked to glucose homeostasis
Thiamine (B1)	Vit. B1 - A	Micronutrient	mg	Continuous	Thiamine deficiency associated with impaired glucose metabolism
Riboflavin (B2)	Vit. B2 - A	Micronutrient	mg	Continuous	Riboflavin involved in glucose and fatty acid metabolism
Vitamin B6	Vit. B6 - A	Micronutrient	mg	Continuous	Pyridoxine role in amino acid metabolism and insulin sensitivity
Vitamin B12	Vit. B12 - A	Micronutrient	μg	Continuous	B12 deficiency linked to insulin resistance and GDM risk
Vitamin C	Vit. C - A	Micronutrient	mg	Continuous	Antioxidant; protective against oxidative stress in GDM
Vitamin D	Vit. D - A	Micronutrient	μg	Continuous	Vitamin D deficiency is a recognized GDM risk factor

Display Name	Column Name	Category	Unit	Type	Clinical Relevance to GDM
Vitamin E	Vit. E - A	Micronutrient	mg	Continuous	α-tocopherol; antioxidant protecting against lipid peroxidation
Vitamin K	Vit. K - A	Micronutrient	µg	Continuous	Emerging evidence for role in glucose metabolism via osteocalcin
Folic Acid (DFE)	folic acid eq. - A	Micronutrient	µg DFE	Continuous	Folate essential for DNA synthesis; deficiency linked to GDM
Niacin	niacine - A	Micronutrient	mg	Continuous	Niacin affects lipid and glucose metabolism
Retinol	retinol - A	Micronutrient	µg	Continuous	Preformed vitamin A; reflects animal-source dietary pattern
Calcium	calcium - A	Micronutrient	mg	Continuous	Calcium supplementation may improve insulin sensitivity
Iron	iron - A	Micronutrient	mg	Continuous	Iron overload associated with increased GDM risk; U-shaped relationship
Zinc	zinc - A	Micronutrient	mg	Continuous	Zinc involved in insulin synthesis, storage, and secretion
Magnesium	magnesium - A	Micronutrient	mg	Continuous	Magnesium deficiency strongly associated with insulin resistance
Phosphorus	phosphorus - A	Micronutrient	mg	Continuous	Phosphorus metabolism linked to glucose regulation
Potassium	potassium - A	Micronutrient	mg	Continuous	Potassium affects insulin secretion and glucose uptake
Sodium	sodium - A	Micronutrient	mg	Continuous	Reflects processed food intake; high sodium linked to metabolic syndrome
Copper	copper - A	Micronutrient	mg	Continuous	Copper involved in antioxidant defense and glucose metabolism
Manganese	manganese - A	Micronutrient	mg	Continuous	Manganese cofactor for mitochondrial antioxidant enzymes
Iodine	iodine - A	Micronutrient	µg	Continuous	Iodine essential for thyroid function; indirect GDM link

All dietary micronutrient features represent absolute intakes from the pre-pregnancy period (up to 6 months before conception), assessed via a validated semi-quantitative Food Frequency Questionnaire (FFQ). Features with the suffix '- A' denote first-trimester data collection timing. BMI: Body Mass Index; ART: Assisted Reproductive Technology; DFE: Dietary Folate Equivalents. Equivalent forms (Vitamin E equivalents, niacin equivalents) were excluded as redundant with their base forms. Note: Entries in

the 'Clinical Relevance to GDM' column reflect putative biological mechanisms reported in the existing literature and should not be interpreted as established causal relationships. The strength of evidence varies considerably across micronutrients, and these annotations are provided for contextual reference only.

Table S6. Comparison of SHAP feature importance rankings between LinearExplainer and KernelExplainer for the Logistic Regression model.

Feature	Rank (Linear)	Mean |SHAP| (Linear)	% (Linear)	Rank (Kernel)	Mean |SHAP| (Kernel)	% (Kernel)	|Rank Δ|
Maternal Age	1	0.4386997573112894	12.352204951249488	1	0.0919662526817833	17.80732731223397	0
Calcium	2	0.2672222490045614	7.524015986395955	2	0.0475239463173514	9.20201098298902	0
Vitamin B6	3	0.2269373117840848	6.389737262274263	4	0.0422410766167051	8.179094563497602	1
Niacin	4	0.2239041461163425	6.304334242658591	3	0.0431811598675623	8.36112187961199	1
Folic Acid	5	0.2111889556065686	5.946320278544488	5	0.039740924061335	7.694992693673222	0
Thiamine (B1)	6	0.1971468089546599	5.550944009218672	7	0.0329378166630628	6.377714271984189	1
Retinol	7	0.1829571296780967	5.151413752601192	8	0.0320122042193012	6.198488922796889	1
Pre-pregnancy BMI	8	0.1817839739343926	5.118381912613904	6	0.0347588430734349	6.730317671471636	2
Magnesium	9	0.1404882729085617	3.955643720546388	9	0.0215836724980511	4.179223460400824	0
Parity	10	0.1331250850139059	3.748322871939647	11	0.0190853911949185	3.6954839191492015	1
Vitamin C	11	0.1310681564842339	3.69040717368556	10	0.0200278147550292	3.877964386845541	1
Assisted Reproductive Technology	12	0.1207507820011693	3.3999070718512763	14	0.0103543897949075	2.0049094403614443	2
Smoking Status	13	0.1165670373114299	3.282107891409304	13	0.0134067287385581	2.595930570966141	0
Manganese	14	0.1096235076811857	3.086603107902001	12	0.0147458764116464	2.8552283050666403	2
Potassium	15	0.0942661239483559	2.654194499915709	15	0.0098952867237145	1.916013802881548	0
Vitamin K	16	0.0905331212204847	2.549086589528789	16	0.0091909078776615	1.7796256790022194	0
Zinc	17	0.0745565824257537	2.0992448052214523	18	0.0048181818107685	0.9329393995326224	1
Copper	18	0.0713558681922559	2.0091242215109566	22	0.0034734538381166	0.6725611579858393	4
Vitamin A	19	0.0672152409356077	1.8925390726740563	23	0.0025437269684062	0.492539136895139	4
Thyroid Condition	20	0.0650300639206399	1.8310123590303808	17	0.0053657879379927	1.0389717892550487	3
Vitamin E	21	0.061939615780182	1.7439964713168696	19	0.0045334014060056	0.8777976738251356	2
Vitamin D	22	0.0603915977371496	1.7004098592499752	20	0.0045277308105682	0.8766996824852231	2
Iodine	23	0.0547637239193202	1.5419492043737208	24	0.0023261840815618	0.4504165399911476	1
Riboflavin (B2)	24	0.049025906160671	1.3803929241465984	25	0.0018062487377117	0.3497420145086921	1
Pre-pregnancy Weight	25	0.0451626723974706	1.2716181769023729	21	0.003502921809031	0.6782670097303376	4
Height	26	0.0353573053454327	0.9955343600538964	26	0.0008640930687785	0.1673134182950113	0

Iron	27	0.0347734958481918	0.9790963875175048	27	3.772326004104383e-05	0.0073043145637317	0
Sodium	28	0.0259539279488227	0.7307691239189353	29	0.0	0.0	1
Phosphorus	29	0.0250117879572321	0.7042418553828874	29	0.0	0.0	0
Vitamin B12	30	0.0123346807607373	0.3473001801930575	29	0.0	0.0	1
Walking Frequency	31	0.0024557713764904	0.0691456761720916	29	0.0	0.0	2

Text S1. Feature Selection Rationale

1. Overview of Available Data

The BORN2020 dataset comprises 1,160 columns of which 205 correspond to first-trimester measurements (identified by the '- A' suffix). These span dietary intake from the Food Frequency Questionnaire (FFQ) processed through NutriSurvey, anthropometric measurements, clinical history, and lifestyle variables.

2. Included Feature Categories

2.1 Demographic Features (4)

Maternal age, pre-pregnancy weight, height, and BMI were included as established GDM risk factors universally available at the first prenatal visit.

2.2 Clinical Features (3)

Assisted reproductive technology (ART), thyroid condition, and smoking status were included based on their documented associations with metabolic function during pregnancy.

2.3 Obstetric Features (1)

Parity was included as a count variable, as multiparity has been associated with GDM recurrence in longitudinal studies.

2.4 Lifestyle Features (1)

Walking frequency (times per week with >10 minutes per session) was retained from the IPAQ-SF as the only lifestyle variable with sufficient variance.

2.5 Dietary Micronutrient Features (22)

Twenty-two absolute vitamin and mineral intakes from the pre-pregnancy period were included. Equivalent forms (Vitamin E equivalents and niacin equivalents) were excluded as computationally derived from base forms already present. Folic acid was retained as dietary folate equivalents (DFE) since no separate base folic acid column was available.

3. Excluded Feature Categories

3.1 Macronutrient Features

Total energy, protein, fat, carbohydrate, and fiber intakes were excluded. Total energy intake was used for the nutrient residual sensitivity analysis (see Section 2.4) but was not included as a model feature. The study's primary hypothesis concerns micronutrient intake patterns. Macronutrient intake was addressed separately in related BORN2020 publications (Tranidou et al., Biomedicines 2024).

3.2 Amino Acid Features (~18)

Individual amino acid intakes were analysed in a dedicated BORN2020 publication (Tranidou et al., 2025, Nutrients 17:173) and excluded to avoid overlap.

3.3 Second-Trimester and Outcome Variables

All second-trimester features ('- B', n=205), difference features (n=33), and outcome variables were strictly excluded to prevent data leakage.

3.4 Biochemical Markers

The BORN2020 dataset does not include first-trimester biochemical markers (fasting glucose, HbA1c, PAPP-A, free β-hCG, triglycerides). Their absence likely contributes to the moderate discriminative performance observed and represents a limitation discussed in the main text.

4. Sample Size Considerations

With 797 participants and 31 features, the events-per-variable ratio for the minority class (117 GDM cases) is approximately 3.8:1. While below the commonly recommended EPV of 10:1 for logistic regression, this is acceptable for regularized and tree-based ensemble methods that incorporate internal regularization mechanisms.

Figure S1. Comparison of SHAP feature importance rankings derived from LinearExplainer and KernelExplainer for the best-performing Logistic Regression model. The left panel shows rank agreement across all 31 features, demonstrating near-perfect concordance (Spearman ρ = 0.982, $p < 0.001$). The right panel displays the top 15 features by relative contribution (%) for both explainers, highlighting consistent ordering and magnitude of feature importance. This analysis confirms the robustness of feature ranking to the choice of SHAP explainer.

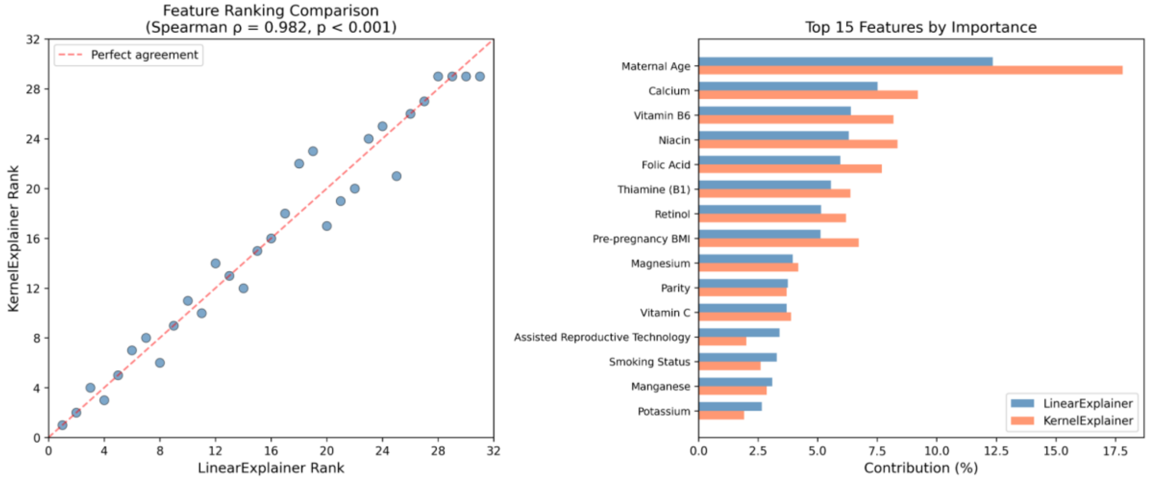

Supplement: Supplementary file 1 [file jcm-15-02461-s001.zip › jcm-4189397-supplementary.pdf]
